# Supplementary material for: Crystal Structure of a Retroviral Polyprotein: Prototype Foamy Virus Protease-Reverse Transcriptase (PR-RT)
Source: Viruses. 2021 Jul 29;13(8):1495. doi: 10.3390/v13081495 (PMC8402755; doi:10.3390/v13081495)
Supplement: Supplementary file 1 [file viruses-13-01495-s001.zip › D_1000253082_val-report-full_P1.pdf]

Full wwPDB X-ray Structure Validation Report (i)

Dec 16, 2020 – 04:50 PM EST

PDB ID	:	7KSF
Title	:	Crystal structure of Prototype Foamy Virus Proteas-Reverse Transcriptase (native)
Deposited on	:	2020-11-21
Resolution	:	2.90 Å(reported)

This is a Full wwPDB X-ray Structure Validation Report.

This report is produced by the wwPDB biocuration pipeline after annotation of the structure.

We welcome your comments at *validation@mail.wwpdb.org*
A user guide is available at
https://www.wwpdb.org/validation/2017/XrayValidationReportHelp
with specific help available everywhere you see the (i) symbol.

CONFIDENTIAL VALIDATION REPORT

The following versions of software and data (see references (i)) were used in the production of this report:

MolProbity	:	4.02b-467
Xtriage (Phenix)	:	1.13
EDS	:	2.15.1
Percentile statistics	:	20191225.v01 (using entries in the PDB archive December 25th 2019)
Refmac	:	5.8.0158
CCP4	:	7.0.044 (Gargrove)
Ideal geometry (proteins)	:	Engh & Huber (2001)
Ideal geometry (DNA, RNA)	:	Parkinson et al. (1996)
Validation Pipeline (wwPDB-VP)	:	2.15.1

1 Overall quality at a glance ⓘ

The following experimental techniques were used to determine the structure:
X-RAY DIFFRACTION

The reported resolution of this entry is 2.90 Å.

Percentile scores (ranging between 0-100) for global validation metrics of the entry are shown in the following graphic. The table shows the number of entries on which the scores are based.

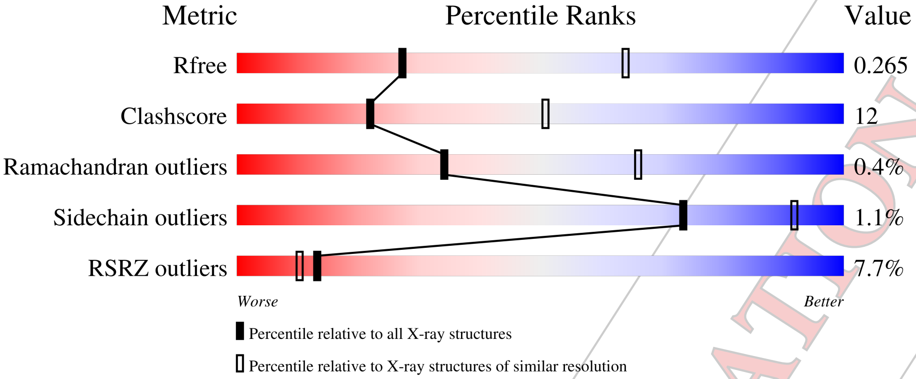

Metric	Whole archive (#Entries)	Similar resolution (#Entries, resolution range(Å))
R_{free}	130704	1957 (2.90-2.90)
Clashscore	141614	2172 (2.90-2.90)
Ramachandran outliers	138981	2115 (2.90-2.90)
Sidechain outliers	138945	2117 (2.90-2.90)
RSRZ outliers	127900	1906 (2.90-2.90)

The table below summarises the geometric issues observed across the polymeric chains and their fit to the electron density. The red, orange, yellow and green segments of the lower bar indicate the fraction of residues that contain outliers for >=3, 2, 1 and 0 types of geometric quality criteria respectively. A grey segment represents the fraction of residues that are not modelled. The numeric value for each fraction is indicated below the corresponding segment, with a dot representing fractions <=5% The upper red bar (where present) indicates the fraction of residues that have poor fit to the electron density. The numeric value is given above the bar.

Mol	Chain	Length	Quality of chain
1	A	747	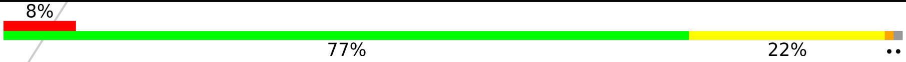

CONFIDENTIAL VALIDATION REPORT

2 Entry composition (i)

There are 3 unique types of molecules in this entry. The entry contains 5910 atoms, of which 0 are hydrogens and 0 are deuteriums.

In the tables below, the ZeroOcc column contains the number of atoms modelled with zero occupancy, the AltConf column contains the number of residues with at least one atom in alternate conformation and the Trace column contains the number of residues modelled with at most 2 atoms.

- Molecule 1 is a protein called Protease/Reverse transcriptase/ribonuclease H.

Mol	Chain	Residues	Atoms					ZeroOcc	AltConf	Trace
1	A	742	Total<br>5901	C<br>3810	N<br>986	O<br>1088	S<br>17	0	0	0

There are 12 discrepancies between the modelled and reference sequences:

Chain	Residue	Modelled	Actual	Comment	Reference
A	26	ALA	ASP	engineered mutation	UNP P14350
A	726	HIS	ILE	conflict	UNP P14350
A	727	GLN	SER	conflict	UNP P14350
A	728	PRO	LEU	conflict	UNP P14350
A	729	THR	GLN	conflict	UNP P14350
A	730	ASN	ILE	conflict	UNP P14350
A	731	THR	PRO	conflict	UNP P14350
A	732	SER	VAL	conflict	UNP P14350
A	733	ILE	PHE	conflict	UNP P14350
A	734	HIS	ILE	conflict	UNP P14350
A	735	THR	LEU	conflict	UNP P14350
A	736	GLU	LYS	conflict	UNP P14350

- Molecule 2 is CALCIUM ION (three-letter code: CA) (formula: Ca).

Mol	Chain	Residues	Atoms		ZeroOcc	AltConf
2	A	1	Total<br>1	Ca<br>1	0	0

- Molecule 3 is water.

Mol	Chain	Residues	Atoms		ZeroOcc	AltConf
3	A	8	Total<br>8	O<br>8	0	0

CONFIDENTIAL VALIDATION REPORT

3 Residue-property plots (i)

These plots are drawn for all protein, RNA, DNA and oligosaccharide chains in the entry. The first graphic for a chain summarises the proportions of the various outlier classes displayed in the second graphic. The second graphic shows the sequence view annotated by issues in geometry and electron density. Residues are color-coded according to the number of geometric quality criteria for which they contain at least one outlier: green = 0, yellow = 1, orange = 2 and red = 3 or more. A red dot above a residue indicates a poor fit to the electron density (RSRZ > 2). Stretches of 2 or more consecutive residues without any outlier are shown as a green connector. Residues present in the sample, but not in the model, are shown in grey.

- Molecule 1: Protease/Reverse transcriptase/ribonuclease H

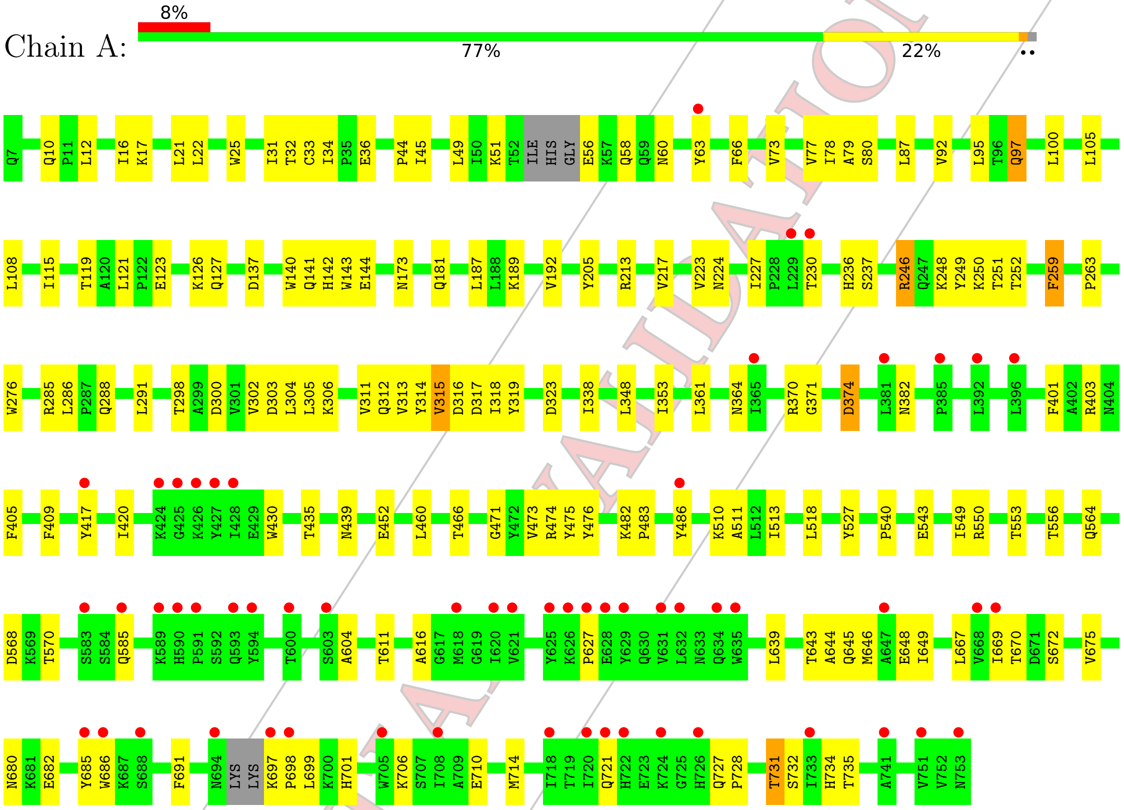

CONFIDENTIAL VALIDATION REPORT

4 Data and refinement statistics

Property	Value	Source
Space group	C 1 2 1	Depositor
Cell constants<br>a, b, c, α, β, γ	228.21Å 52.54Å 75.43Å<br>90.00° 90.09° 90.00°	Depositor
Resolution (Å)	50.00 – 2.90<br>75.43 – 2.90	Depositor<br>EDS
% Data completeness<br>(in resolution range)	96.1 (50.00-2.90)<br>90.1 (75.43-2.90)	Depositor<br>EDS
R_{merge}	0.13	Depositor
R_{sym}	(Not available)	Depositor
$< I/\sigma(I) >$ [1]	0.83 (at 2.91Å)	Xtriage
Refinement program	PHENIX 1.18.2_3874	Depositor
R, R_{free}	0.225 , 0.257<br>0.223 , 0.265	Depositor<br>DCC
R_{free} test set	971 reflections (4.99%)	wwPDB-VP
Wilson B-factor (Å^2)	79.6	Xtriage
Anisotropy	0.433	Xtriage
Bulk solvent k_{sol}(e/Å^3), B_{sol}(Å^2)	0.32 , 58.0	EDS
L-test for twinning[2]	$< \|L\| >= 0.38$, $< L^2 >= 0.21$	Xtriage
Estimated twinning fraction	0.409 for -h,-k,l	Xtriage
F_o,F_c correlation	0.93	EDS
Total number of atoms	5910	wwPDB-VP
Average B, all atoms (Å^2)	103.0	wwPDB-VP

Xtriage's analysis on translational NCS is as follows: *The largest off-origin peak in the Patterson function is 4.23% of the height of the origin peak. No significant pseudotranslation is detected.*

[1] Intensities estimated from amplitudes.

[2] Theoretical values of $< |L| >$, $< L^2 >$ for acentric reflections are 0.5, 0.333 respectively for untwinned datasets, and 0.375, 0.2 for perfectly twinned datasets.

CONFIDENTIAL VALIDATION REPORT

5 Model quality (i)

5.1 Standard geometry (i)

Bond lengths and bond angles in the following residue types are not validated in this section: CA

The Z score for a bond length (or angle) is the number of standard deviations the observed value is removed from the expected value. A bond length (or angle) with $|Z| > 5$ is considered an outlier worth inspection. RMSZ is the root-mean-square of all Z scores of the bond lengths (or angles).

Mol	Chain	Bond lengths		Bond angles	
		RMSZ	#$\|Z\|$ >5	RMSZ	#$\|Z\|$ >5
1	A	0.30	0/6051	0.44	0/8243

There are no bond length outliers.

There are no bond angle outliers.

There are no chirality outliers.

There are no planarity outliers.

5.2 Too-close contacts (i)

In the following table, the Non-H and H(model) columns list the number of non-hydrogen atoms and hydrogen atoms in the chain respectively. The H(added) column lists the number of hydrogen atoms added and optimized by MolProbity. The Clashes column lists the number of clashes within the asymmetric unit, whereas Symm-Clashes lists symmetry-related clashes.

Mol	Chain	Non-H	H(model)	H(added)	Clashes	Symm-Clashes
1	A	5901	0	5939	140	0
2	A	1	0	0	0	0
3	A	8	0	0	2	0
All	All	5910	0	5939	140	0

The all-atom clashscore is defined as the number of clashes found per 1000 atoms (including hydrogen atoms). The all-atom clashscore for this structure is 12.

All (140) close contacts within the same asymmetric unit are listed below, sorted by their clash magnitude.

Atom-1	Atom-2	Interatomic distance (Å)	Clash overlap (Å)
1:A:319:TYR:CD2	1:A:361:LEU:HD22	1.71	1.25

Continued on next page...

CONFIDENTIAL VALIDATION REPORT

Continued from previous page...

Atom-1	Atom-2	Interatomic distance (Å)	Clash overlap (Å)
1:A:51:LYS:HA	1:A:56:GLU:HG2	1.15	1.06
1:A:303:ASP:HA	1:A:306:LYS:HE2	1.52	0.89
1:A:305:LEU:HD12	1:A:313:VAL:HG21	1.57	0.87
1:A:697:LYS:N	1:A:698:PRO:CD	2.39	0.86
1:A:319:TYR:CD2	1:A:361:LEU:CD2	2.56	0.86
1:A:51:LYS:CA	1:A:56:GLU:HG2	2.04	0.85
1:A:246:ARG:HH21	1:A:371:GLY:HA3	1.41	0.85
1:A:31:ILE:HD11	1:A:78:ILE:HG23	1.58	0.85
1:A:315:VAL:HG12	1:A:316:ASP:H	1.41	0.84
1:A:300:ASP:O	1:A:304:LEU:HD13	1.84	0.78
1:A:302:VAL:O	1:A:306:LYS:HG3	1.84	0.77
1:A:319:TYR:CE2	1:A:361:LEU:HD22	2.19	0.76
1:A:227:ILE:HG22	1:A:291:LEU:HD23	1.69	0.74
1:A:246:ARG:HH21	1:A:371:GLY:CA	2.02	0.72
1:A:33:CYS:HB3	1:A:80:SER:HB2	1.71	0.72
1:A:303:ASP:HA	1:A:306:LYS:CE	2.19	0.71
1:A:315:VAL:HG12	1:A:316:ASP:N	2.04	0.71
1:A:319:TYR:HB2	1:A:361:LEU:HD11	1.72	0.70
1:A:305:LEU:HD12	1:A:313:VAL:CG2	2.21	0.70
1:A:305:LEU:HD13	1:A:311:VAL:HG21	1.73	0.70
1:A:319:TYR:HB2	1:A:361:LEU:CD1	2.22	0.69
1:A:697:LYS:N	1:A:698:PRO:HD3	2.07	0.69
1:A:643:THR:HB	1:A:646:MET:HB2	1.74	0.69
1:A:731:THR:HG23	1:A:735:THR:HG21	1.75	0.69
1:A:246:ARG:HG2	1:A:250:LYS:HE2	1.76	0.68
1:A:430:TRP:HE1	1:A:435:THR:HG1	0.71	0.67
1:A:303:ASP:CA	1:A:306:LYS:HE2	2.25	0.66
1:A:251:THR:HG23	1:A:353:ILE:HA	1.79	0.64
1:A:303:ASP:HA	1:A:306:LYS:CD	2.28	0.64
1:A:119:THR:HG22	1:A:121:LEU:H	1.62	0.64
1:A:305:LEU:CD1	1:A:313:VAL:HG21	2.29	0.62
1:A:364:ASN:HB3	1:A:550:ARG:HH22	1.65	0.62
1:A:364:ASN:HB3	1:A:550:ARG:NH2	2.15	0.60
1:A:304:LEU:HB3	1:A:338:ILE:HG21	1.83	0.60
1:A:670:THR:HG22	1:A:672:SER:H	1.67	0.60
1:A:173:ASN:N	3:A:901:HOH:O	2.35	0.59
1:A:645:GLN:O	1:A:649:ILE:N	2.35	0.59
1:A:87:LEU:HD21	1:A:92:VAL:HG21	1.84	0.59
1:A:731:THR:CG2	1:A:735:THR:HG21	2.32	0.59
1:A:361:LEU:HG	1:A:361:LEU:O	2.01	0.58
1:A:12:LEU:HD13	1:A:95:LEU:HD21	1.85	0.57

Continued on next page...

CONFIDENTIAL VALIDATION REPORT

Continued from previous page...

Atom-1	Atom-2	Interatomic distance (Å)	Clash overlap (Å)
1:A:298:THR:O	1:A:302:VAL:HG23	2.04	0.57
1:A:452:GLU:HB2	1:A:482:LYS:HB2	1.88	0.55
1:A:476:TYR:CZ	1:A:483:PRO:HG3	2.40	0.55
1:A:611:THR:HG22	3:A:907:HOH:O	2.07	0.55
1:A:697:LYS:N	1:A:698:PRO:HD2	2.22	0.55
1:A:305:LEU:CD1	1:A:313:VAL:CG2	2.85	0.55
1:A:643:THR:HG22	1:A:645:GLN:H	1.72	0.55
1:A:403:ARG:HG2	1:A:409:PHE:CE2	2.41	0.55
1:A:36:GLU:HG2	1:A:79:ALA:HB1	1.89	0.54
1:A:144:GLU:OE1	1:A:213:ARG:NH2	2.40	0.54
1:A:304:LEU:N	1:A:304:LEU:HD12	2.23	0.54
1:A:10:GLN:HB2	1:A:100:LEU:HA	1.89	0.54
1:A:236:HIS:ND1	1:A:556:THR:OG1	2.41	0.54
1:A:311:VAL:HG22	1:A:312:GLN:N	2.22	0.53
1:A:382:ASN:ND2	1:A:439:ASN:OD1	2.39	0.53
1:A:303:ASP:HA	1:A:306:LYS:HG3	1.90	0.52
1:A:473:VAL:HG21	1:A:511:ALA:HB2	1.92	0.52
1:A:460:LEU:HD11	1:A:518:LEU:HB2	1.92	0.52
1:A:669:ILE:HG12	1:A:734:HIS:CE1	2.45	0.51
1:A:224:ASN:HB3	1:A:291:LEU:HD13	1.92	0.51
1:A:540:PRO:HB2	1:A:543:GLU:HG3	1.92	0.51
1:A:316:ASP:OD1	1:A:317:ASP:N	2.44	0.51
1:A:246:ARG:NH2	1:A:371:GLY:HA3	2.20	0.51
1:A:49:LEU:HD12	1:A:49:LEU:C	2.31	0.51
1:A:527:TYR:HB3	1:A:568:ASP:HB2	1.92	0.51
1:A:646:MET:HA	1:A:649:ILE:HB	1.91	0.51
1:A:49:LEU:O	1:A:49:LEU:HD12	2.12	0.50
1:A:403:ARG:NH2	1:A:417:TYR:OH	2.44	0.50
1:A:230:THR:OG1	1:A:230:THR:O	2.30	0.49
1:A:44:PRO:HB3	1:A:60:ASN:HB3	1.94	0.49
1:A:108:LEU:HD11	1:A:348:LEU:HD12	1.94	0.49
1:A:259:PHE:CE1	1:A:288:GLN:HB3	2.47	0.49
1:A:304:LEU:CD1	1:A:304:LEU:N	2.76	0.48
1:A:549:ILE:HD12	1:A:549:ILE:H	1.78	0.48
1:A:123:GLU:HA	1:A:126:LYS:HB3	1.96	0.47
1:A:417:TYR:O	1:A:420:ILE:HG12	2.14	0.47
1:A:732:SER:O	1:A:735:THR:HG22	2.14	0.47
1:A:25:TRP:CD2	1:A:97:GLN:HB2	2.49	0.47
1:A:246:ARG:HB3	1:A:370:ARG:HA	1.97	0.46
1:A:319:TYR:HD2	1:A:361:LEU:HD22	1.63	0.46
1:A:483:PRO:HG2	1:A:486:TYR:CE2	2.50	0.46

Continued on next page...

CONFIDENTIAL VALIDATION REPORT

Continued from previous page...

Atom-1	Atom-2	Interatomic distance (Å)	Clash overlap (Å)
1:A:648:GLU:OE1	1:A:672:SER:HB2	2.15	0.46
1:A:311:VAL:O	1:A:312:GLN:CD	2.54	0.46
1:A:263:PRO:HA	1:A:285:ARG:HA	1.97	0.46
1:A:483:PRO:HG2	1:A:486:TYR:CZ	2.51	0.46
1:A:305:LEU:HD13	1:A:311:VAL:CG2	2.44	0.46
1:A:691:PHE:CE2	1:A:706:LYS:HG2	2.51	0.46
1:A:303:ASP:HA	1:A:306:LYS:CG	2.46	0.46
1:A:319:TYR:HB2	1:A:361:LEU:HD13	1.97	0.45
1:A:405:PHE:CG	1:A:482:LYS:HG2	2.51	0.45
1:A:401:PHE:HE2	1:A:486:TYR:HH	1.64	0.45
1:A:474:ARG:HG2	1:A:486:TYR:HD2	1.81	0.45
1:A:34:ILE:HD12	1:A:77:VAL:HG21	1.98	0.45
1:A:181:GLN:OE1	1:A:276:TRP:NE1	2.41	0.45
1:A:727:GLN:N	1:A:728:PRO:HD3	2.31	0.45
1:A:187:LEU:HB3	1:A:192:VAL:HB	1.99	0.45
1:A:16:ILE:HG23	1:A:17:LYS:H	1.82	0.45
1:A:259:PHE:O	1:A:286:LEU:HB2	2.16	0.45
1:A:115:ILE:O	1:A:119:THR:OG1	2.30	0.44
1:A:248:LYS:H	1:A:323:ASP:HA	1.81	0.44
1:A:374:ASP:N	1:A:374:ASP:OD2	2.50	0.44
1:A:66:PHE:O	1:A:73:VAL:N	2.49	0.44
1:A:728:PRO:HB2	1:A:731:THR:HG22	2.00	0.44
1:A:237:SER:HB2	1:A:553:THR:HG23	2.00	0.43
1:A:405:PHE:HB2	1:A:482:LYS:HE2	1.99	0.43
1:A:731:THR:HG23	1:A:735:THR:CG2	2.47	0.43
1:A:49:LEU:HA	1:A:58:GLN:HA	1.99	0.43
1:A:568:ASP:OD1	1:A:570:THR:HG22	2.17	0.43
1:A:616:ALA:HB3	1:A:639:LEU:HB2	2.01	0.43
1:A:314:TYR:CD2	1:A:315:VAL:HG23	2.54	0.43
1:A:32:THR:HB	1:A:77:VAL:HG12	2.00	0.43
1:A:585:GLN:HG3	1:A:680:ASN:HB3	2.00	0.42
1:A:34:ILE:O	1:A:80:SER:N	2.52	0.42
1:A:223:VAL:HG12	1:A:227:ILE:HD13	2.02	0.42
1:A:682:GLU:HB3	1:A:686:TRP:CD1	2.55	0.42
1:A:305:LEU:HB3	1:A:311:VAL:HG21	2.02	0.42
1:A:466:THR:HA	1:A:471:GLY:HA2	2.02	0.42
1:A:604:ALA:HB3	1:A:644:ALA:HB2	2.02	0.42
1:A:123:GLU:OE2	1:A:127:GLN:NE2	2.35	0.41
1:A:121:LEU:HD23	1:A:249:TYR:CE2	2.55	0.41
1:A:252:THR:HA	1:A:318:ILE:O	2.20	0.41
1:A:667:LEU:HD11	1:A:721:GLN:HG3	2.02	0.41

Continued on next page...

CONFIDENTIAL VALIDATION REPORT

Continued from previous page...

Atom-1	Atom-2	Interatomic distance (Å)	Clash overlap (Å)
1:A:10:GLN:OE1	1:A:189:LYS:HE2	2.20	0.41
1:A:259:PHE:CZ	1:A:288:GLN:HB3	2.55	0.41
1:A:475:TYR:OH	1:A:511:ALA:O	2.27	0.41
1:A:137:ASP:HA	1:A:140:TRP:CD1	2.55	0.41
1:A:141:GLN:NE2	1:A:143:TRP:O	2.54	0.41
1:A:45:ILE:HD11	1:A:63:TYR:HB2	2.01	0.41
1:A:686:TRP:HE3	1:A:691:PHE:HA	1.85	0.41
1:A:697:LYS:N	1:A:701:HIS:HB3	2.35	0.41
1:A:21:LEU:HG	1:A:22:LEU:H	1.85	0.41
1:A:670:THR:HG21	1:A:675:VAL:HB	2.03	0.41
1:A:710:GLU:O	1:A:714:MET:HG2	2.21	0.41
1:A:105:LEU:HD12	1:A:142:HIS:O	2.21	0.41
1:A:304:LEU:HB3	1:A:338:ILE:CG2	2.49	0.41
1:A:510:LYS:HA	1:A:513:ILE:HD12	2.02	0.40
1:A:205:TYR:O	1:A:217:VAL:N	2.49	0.40
1:A:669:ILE:HG12	1:A:734:HIS:NE2	2.37	0.40

There are no symmetry-related clashes.

5.3 Torsion angles (i)

5.3.1 Protein backbone (i)

In the following table, the Percentiles column shows the percent Ramachandran outliers of the chain as a percentile score with respect to all X-ray entries followed by that with respect to entries of similar resolution.

The Analysed column shows the number of residues for which the backbone conformation was analysed, and the total number of residues.

Mol	Chain	Analysed	Favoured	Allowed	Outliers	Percentiles
1	A	736/747 (98%)	693 (94%)	40 (5%)	3 (0%)	34 66

All (3) Ramachandran outliers are listed below:

Mol	Chain	Res	Type
1	A	315	VAL
1	A	699	LEU
1	A	627	PRO

CONFIDENTIAL VALIDATION REPORT

5.3.2 Protein sidechains (i)

In the following table, the Percentiles column shows the percent sidechain outliers of the chain as a percentile score with respect to all X-ray entries followed by that with respect to entries of similar resolution.

The Analysed column shows the number of residues for which the sidechain conformation was analysed, and the total number of residues.

Mol	Chain	Analysed	Rotameric	Outliers	Percentiles	
1	A	649/669 (97%)	642 (99%)	7 (1%)	73	92

All (7) residues with a non-rotameric sidechain are listed below:

Mol	Chain	Res	Type
1	A	97	GLN
1	A	246	ARG
1	A	259	PHE
1	A	374	ASP
1	A	564	GLN
1	A	685	TYR
1	A	731	THR

Sometimes sidechains can be flipped to improve hydrogen bonding and reduce clashes. All (1) such sidechains are listed below:

Mol	Chain	Res	Type
1	A	262	HIS

5.3.3 RNA (i)

There are no RNA molecules in this entry.

5.4 Non-standard residues in protein, DNA, RNA chains (i)

There are no non-standard protein/DNA/RNA residues in this entry.

5.5 Carbohydrates (i)

There are no monosaccharides in this entry.

CONFIDENTIAL VALIDATION REPORT

5.6 Ligand geometry (i)

Of 1 ligands modelled in this entry, 1 is monoatomic - leaving 0 for Mogul analysis.

There are no bond length outliers.

There are no bond angle outliers.

There are no chirality outliers.

There are no torsion outliers.

There are no ring outliers.

No monomer is involved in short contacts.

5.7 Other polymers (i)

There are no such residues in this entry.

5.8 Polymer linkage issues (i)

There are no chain breaks in this entry.

CONFIDENTIAL VALIDATION REPORT

6 Fit of model and data (i)

6.1 Protein, DNA and RNA chains (i)

In the following table, the column labelled '#RSRZ> 2' contains the number (and percentage) of RSRZ outliers, followed by percent RSRZ outliers for the chain as percentile scores relative to all X-ray entries and entries of similar resolution. The OWAB column contains the minimum, median, 95th percentile and maximum values of the occupancy-weighted average B-factor per residue. The column labelled 'Q< 0.9' lists the number of (and percentage) of residues with an average occupancy less than 0.9.

Mol	Chain	Analysed	<RSRZ>	#RSRZ>2			OWAB(Å^2)	Q<0.9
1	A	742/747 (99%)	0.48	57 (7%)	13	10	54, 100, 153, 182	0

All (57) RSRZ outliers are listed below:

Mol	Chain	Res	Type	RSRZ
1	A	694	ASN	6.2
1	A	626	LYS	5.1
1	A	720	ILE	4.9
1	A	585	GLN	4.6
1	A	685	TYR	4.3
1	A	686	TRP	3.9
1	A	721	GLN	3.8
1	A	635	TRP	3.7
1	A	590	HIS	3.7
1	A	486	TYR	3.6
1	A	631	VAL	3.6
1	A	365	ILE	3.6
1	A	718	ILE	3.6
1	A	620	ILE	3.6
1	A	668	VAL	3.5
1	A	647	ALA	3.4
1	A	426	LYS	3.4
1	A	741	ALA	3.4
1	A	698	PRO	3.3
1	A	697	LYS	3.3
1	A	669	ILE	3.1
1	A	229	LEU	3.1
1	A	230	THR	3.0
1	A	724	LYS	3.0
1	A	627	PRO	2.9
1	A	600	THR	2.9
1	A	417	TYR	2.9

Continued on next page...

CONFIDENTIAL VALIDATION REPORT

Continued from previous page...

Mol	Chain	Res	Type	RSRZ
1	A	427	TYR	2.8
1	A	392	LEU	2.8
1	A	688	SER	2.7
1	A	634	GLN	2.7
1	A	726	HIS	2.6
1	A	424	LYS	2.6
1	A	705	TRP	2.6
1	A	591	PRO	2.6
1	A	589	LYS	2.6
1	A	396	LEU	2.4
1	A	628	GLU	2.4
1	A	593	GLN	2.4
1	A	428	ILE	2.4
1	A	632	LEU	2.3
1	A	603	SER	2.3
1	A	621	VAL	2.3
1	A	722	HIS	2.3
1	A	629	TYR	2.3
1	A	618	MET	2.2
1	A	751	VAL	2.2
1	A	385	PRO	2.2
1	A	625	TYR	2.2
1	A	381	LEU	2.1
1	A	753	ASN	2.1
1	A	583	SER	2.1
1	A	63	TYR	2.1
1	A	594	TYR	2.1
1	A	425	GLY	2.0
1	A	733	ILE	2.0
1	A	708	ILE	2.0

6.2 Non-standard residues in protein, DNA, RNA chains (i)

There are no non-standard protein/DNA/RNA residues in this entry.

6.3 Carbohydrates (i)

There are no monosaccharides in this entry.

CONFIDENTIAL VALIDATION REPORT

6.4 Ligands ⓘ

In the following table, the Atoms column lists the number of modelled atoms in the group and the number defined in the chemical component dictionary. The B-factors column lists the minimum, median, 95th percentile and maximum values of B factors of atoms in the group. The column labelled 'Q< 0.9' lists the number of atoms with occupancy less than 0.9.

Mol	Type	Chain	Res	Atoms	RSCC	RSR	B-factors($Å^2$)	Q<0.9
2	CA	A	801	1/1	0.69	0.12	138,138,138,138	0

6.5 Other polymers ⓘ

There are no such residues in this entry.

CONFIDENTIAL VALIDATION REPORT
