# Supplementary material for: Crystal Structure of a Retroviral Polyprotein: Prototype Foamy Virus Protease-Reverse Transcriptase (PR-RT)
Source: Viruses. 2021 Jul 29;13(8):1495. doi: 10.3390/v13081495 (PMC8402755; doi:10.3390/v13081495)
Supplement: Supplementary file 1 [file viruses-13-01495-s001.zip › D_1000253110_val-report-full_P1.pdf]

Full wwPDB X-ray Structure Validation Report (i)

Jan 5, 2021 – 01:34 PM EST

PDB ID	:	7KSE
Title	:	Crystal structure of Prototype Foamy Virus Proteas-Reverse Transcriptase CSH mutant (selenomethionine-labeled)
Deposited on	:	2020-11-21
Resolution	:	3.00 Å(reported)

This is a Full wwPDB X-ray Structure Validation Report.

This report is produced by the wwPDB biocuration pipeline after annotation of the structure.

We welcome your comments at *validation@mail.wwpdb.org*
A user guide is available at
https://www.wwpdb.org/validation/2017/XrayValidationReportHelp
with specific help available everywhere you see the (i) symbol.

CONFIDENTIAL VALIDATION REPORT

The following versions of software and data (see references (i)) were used in the production of this report:

MolProbity	:	4.02b-467
Mogul	:	1.8.5 (274361), CSD as541be (2020)
Xtriage (Phenix)	:	1.13
EDS	:	2.16
Percentile statistics	:	20191225.v01 (using entries in the PDB archive December 25th 2019)
Refmac	:	5.8.0158
CCP4	:	7.0.044 (Gargrove)
Ideal geometry (proteins)	:	Engh & Huber (2001)
Ideal geometry (DNA, RNA)	:	Parkinson et al. (1996)
Validation Pipeline (wwPDB-VP)	:	2.16

1 Overall quality at a glance (i)

The following experimental techniques were used to determine the structure:
X-RAY DIFFRACTION

The reported resolution of this entry is 3.00 Å.

Percentile scores (ranging between 0-100) for global validation metrics of the entry are shown in the following graphic. The table shows the number of entries on which the scores are based.

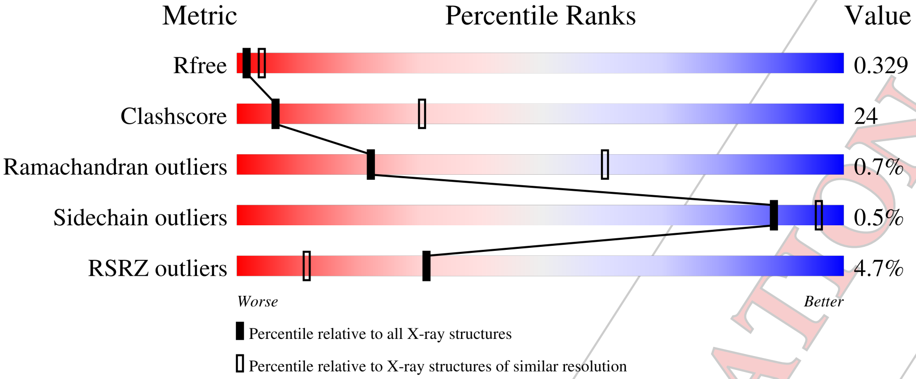

Metric	Whole archive (#Entries)	Similar resolution (#Entries, resolution range(Å))
R_{free}	130704	2092 (3.00-3.00)
Clashscore	141614	2416 (3.00-3.00)
Ramachandran outliers	138981	2333 (3.00-3.00)
Sidechain outliers	138945	2336 (3.00-3.00)
RSRZ outliers	127900	1990 (3.00-3.00)

The table below summarises the geometric issues observed across the polymeric chains and their fit to the electron density. The red, orange, yellow and green segments of the lower bar indicate the fraction of residues that contain outliers for >=3, 2, 1 and 0 types of geometric quality criteria respectively. A grey segment represents the fraction of residues that are not modelled. The numeric value for each fraction is indicated below the corresponding segment, with a dot representing fractions <=5% The upper red bar (where present) indicates the fraction of residues that have poor fit to the electron density. The numeric value is given above the bar.

Mol	Chain	Length	Quality of chain
1	A	747	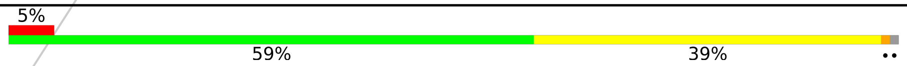

The following table lists non-polymeric compounds, carbohydrate monomers and non-standard residues in protein, DNA, RNA chains that are outliers for geometric or electron-density-fit criteria:

Mol	Type	Chain	Res	Chirality	Geometry	Clashes	Electron density
2	CA	A	801	-	-	-	X

CONFIDENTIAL VALIDATION REPORT

2 Entry composition (i)

There are 3 unique types of molecules in this entry. The entry contains 5908 atoms, of which 0 are hydrogens and 0 are deuteriums.

In the tables below, the ZeroOcc column contains the number of atoms modelled with zero occupancy, the AltConf column contains the number of residues with at least one atom in alternate conformation and the Trace column contains the number of residues modelled with at most 2 atoms.

- Molecule 1 is a protein called Protease/Reverse transcriptase/ribonuclease H.

Mol	Chain	Residues	Atoms						ZeroOcc	AltConf	Trace
			Total	C	N	O	S	Se			
1	A	742	5896	3806	984	1090	3	13	0	0	0

There are 15 discrepancies between the modelled and reference sequences:

Chain	Residue	Modelled	Actual	Comment	Reference
A	26	ALA	ASP	engineered mutation	UNP P14350
A	282	SER	CYS	engineered mutation	UNP P14350
A	509	ASP	HIS	engineered mutation	UNP P14350
A	586	LYS	SER	engineered mutation	UNP P14350
A	726	HIS	ILE	conflict	UNP P14350
A	727	GLN	SER	conflict	UNP P14350
A	728	PRO	LEU	conflict	UNP P14350
A	729	THR	GLN	conflict	UNP P14350
A	730	ASN	ILE	conflict	UNP P14350
A	731	THR	PRO	conflict	UNP P14350
A	732	SER	VAL	conflict	UNP P14350
A	733	ILE	PHE	conflict	UNP P14350
A	734	HIS	ILE	conflict	UNP P14350
A	735	THR	LEU	conflict	UNP P14350
A	736	GLU	LYS	conflict	UNP P14350

- Molecule 2 is CALCIUM ION (three-letter code: CA) (formula: Ca).

Mol	Chain	Residues	Atoms		ZeroOcc	AltConf
			Total	Ca		
2	A	1	1	1	0	0

- Molecule 3 is water.

CONFIDENTIAL VALIDATION REPORT

Mol	Chain	Residues	Atoms		ZeroOcc	AltConf
			Total	O		
3	A	11	11	11	0	0

CONFIDENTIAL VALIDATION REPORT

3 Residue-property plots (i)

These plots are drawn for all protein, RNA, DNA and oligosaccharide chains in the entry. The first graphic for a chain summarises the proportions of the various outlier classes displayed in the second graphic. The second graphic shows the sequence view annotated by issues in geometry and electron density. Residues are color-coded according to the number of geometric quality criteria for which they contain at least one outlier: green = 0, yellow = 1, orange = 2 and red = 3 or more. A red dot above a residue indicates a poor fit to the electron density (RSRZ > 2). Stretches of 2 or more consecutive residues without any outlier are shown as a green connector. Residues present in the sample, but not in the model, are shown in grey.

- Molecule 1: Protease/Reverse transcriptase/ribonuclease H

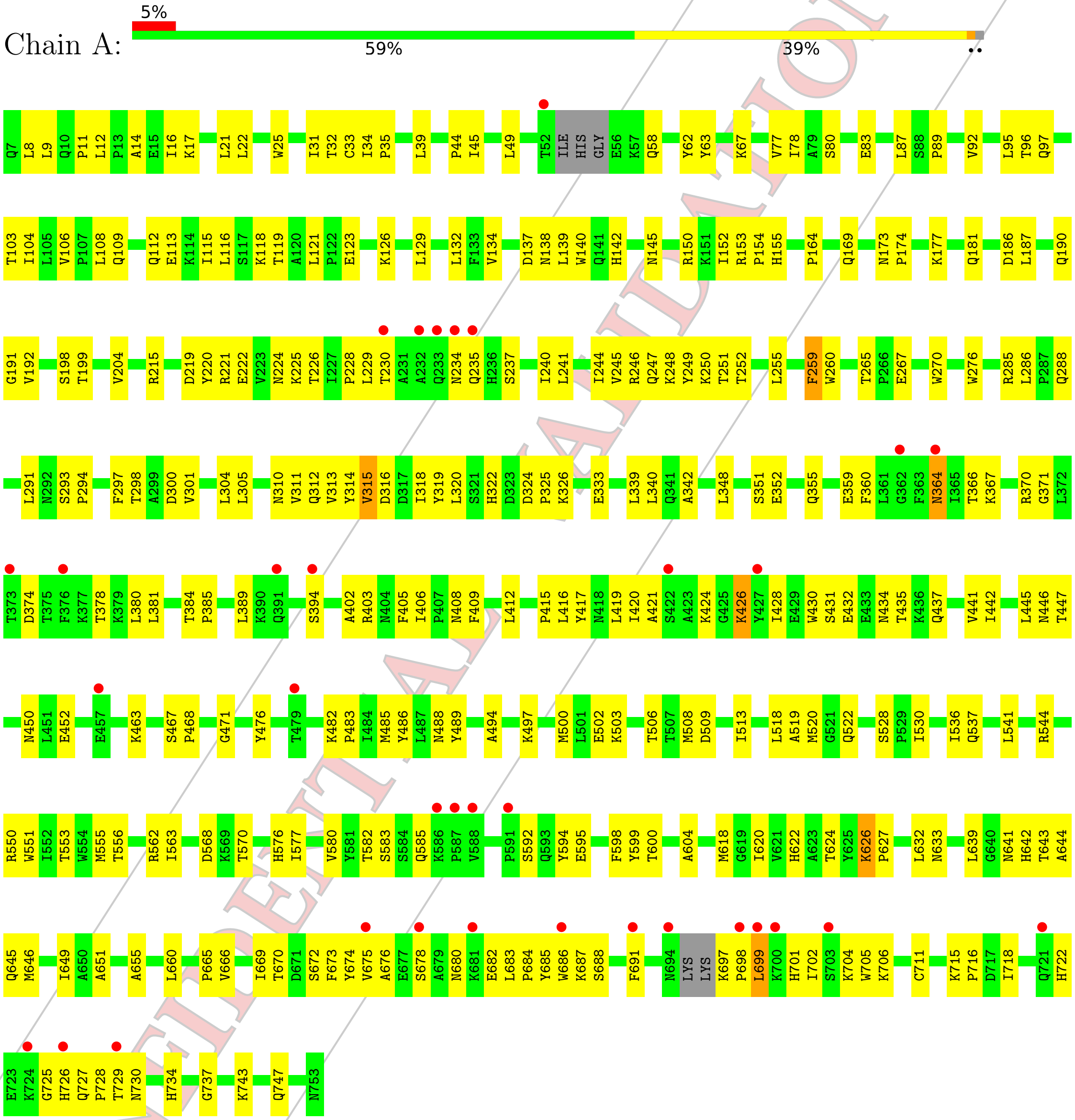

CONFIDENTIAL VALIDATION REPORT

4 Data and refinement statistics (i)

Property	Value	Source
Space group	C 1 2 1	Depositor
Cell constants<br>a, b, c, α, β, γ	240.38Å 53.35Å 74.92Å<br>90.00° 100.00° 90.00°	Depositor
Resolution (Å)	50.00 – 3.00<br>73.78 – 3.00	Depositor<br>EDS
% Data completeness<br>(in resolution range)	99.8 (50.00-3.00)<br>97.8 (73.78-3.00)	Depositor<br>EDS
R_{merge}	0.33	Depositor
R_{sym}	(Not available)	Depositor
$< I/\sigma(I) >$ [1]	2.25 (at 3.01Å)	Xtriage
Refinement program	PHENIX 1.18.2_3874	Depositor
R, R_{free}	0.278 , 0.313<br>0.286 , 0.329	Depositor<br>DCC
R_{free} test set	975 reflections (5.11%)	wwPDB-VP
Wilson B-factor (Å^2)	73.3	Xtriage
Anisotropy	0.532	Xtriage
Bulk solvent k_{sol}(e/Å^3), B_{sol}(Å^2)	0.32 , 51.5	EDS
L-test for twinning[2]	$< \|L\| > = 0.47$, $< L^2 > = 0.29$	Xtriage
Estimated twinning fraction	No twinning to report.	Xtriage
F_o,F_c correlation	0.88	EDS
Total number of atoms	5908	wwPDB-VP
Average B, all atoms (Å^2)	86.0	wwPDB-VP

Xtriage's analysis on translational NCS is as follows: *The largest off-origin peak in the Patterson function is 4.54% of the height of the origin peak. No significant pseudotranslation is detected.*

[1] Intensities estimated from amplitudes.

[2] Theoretical values of $< |L| >$, $< L^2 >$ for acentric reflections are 0.5, 0.333 respectively for untwinned datasets, and 0.375, 0.2 for perfectly twinned datasets.

CONFIDENTIAL VALIDATION REPORT

5 Model quality (i)

5.1 Standard geometry (i)

Bond lengths and bond angles in the following residue types are not validated in this section: CA

The Z score for a bond length (or angle) is the number of standard deviations the observed value is removed from the expected value. A bond length (or angle) with $|Z| > 5$ is considered an outlier worth inspection. RMSZ is the root-mean-square of all Z scores of the bond lengths (or angles).

Mol	Chain	Bond lengths		Bond angles	
		RMSZ	#|Z| >5	RMSZ	#|Z| >5
1	A	0.37	0/6032	0.71	0/8197

There are no bond length outliers.

There are no bond angle outliers.

There are no chirality outliers.

There are no planarity outliers.

5.2 Too-close contacts (i)

In the following table, the Non-H and H(model) columns list the number of non-hydrogen atoms and hydrogen atoms in the chain respectively. The H(added) column lists the number of hydrogen atoms added and optimized by MolProbity. The Clashes column lists the number of clashes within the asymmetric unit, whereas Symm-Clashes lists symmetry-related clashes.

Mol	Chain	Non-H	H(model)	H(added)	Clashes	Symm-Clashes
1	A	5896	0	5929	285	0
2	A	1	0	0	0	0
3	A	11	0	0	5	0
All	All	5908	0	5929	285	0

The all-atom clashscore is defined as the number of clashes found per 1000 atoms (including hydrogen atoms). The all-atom clashscore for this structure is 24.

All (285) close contacts within the same asymmetric unit are listed below, sorted by their clash magnitude.

Atom-1	Atom-2	Interatomic distance (Å)	Clash overlap (Å)
1:A:229:LEU:HD21	1:A:291:LEU:CD1	1.63	1.27

Continued on next page...

CONFIDENTIAL VALIDATION REPORT

Continued from previous page...

Atom-1	Atom-2	Interatomic distance (Å)	Clash overlap (Å)
1:A:406:ILE:HD11	1:A:445:LEU:CD2	1.75	1.16
1:A:406:ILE:HD11	1:A:445:LEU:HD21	1.23	1.10
1:A:402:ALA:HB1	1:A:406:ILE:HD12	1.29	1.08
1:A:626:LYS:CB	1:A:627:PRO:HD3	1.85	1.07
1:A:137:ASP:HA	1:A:140:TRP:CD1	1.95	1.02
1:A:229:LEU:CD2	1:A:291:LEU:HD11	1.89	1.02
1:A:715:LYS:HD3	1:A:718:ILE:HD11	1.44	0.99
1:A:432:GLU:O	1:A:435:THR:HG22	1.63	0.97
1:A:139:LEU:HD23	1:A:139:LEU:O	1.64	0.97
1:A:174:PRO:HA	1:A:177:LYS:HG3	1.48	0.96
1:A:626:LYS:HB3	1:A:627:PRO:HD3	1.45	0.95
1:A:592:SER:HB3	3:A:911:HOH:O	1.67	0.94
1:A:406:ILE:CD1	1:A:445:LEU:HD21	2.00	0.92
1:A:246:ARG:HB2	1:A:371:GLY:O	1.69	0.92
1:A:229:LEU:HD21	1:A:291:LEU:HD11	0.94	0.92
1:A:9:LEU:HD22	1:A:97:GLN:HE21	1.35	0.91
1:A:666:VAL:HG23	1:A:718:ILE:HG23	1.53	0.90
1:A:626:LYS:CB	1:A:627:PRO:CD	2.51	0.87
1:A:119:THR:HG22	1:A:121:LEU:H	1.40	0.86
1:A:137:ASP:HA	1:A:140:TRP:HD1	1.40	0.86
1:A:697:LYS:N	1:A:698:PRO:CD	2.39	0.85
1:A:31:ILE:HD11	1:A:78:ILE:HG23	1.57	0.85
1:A:468:PRO:HG3	1:A:500:MSE:HE1	1.60	0.83
1:A:626:LYS:HB2	1:A:627:PRO:HD3	1.58	0.83
1:A:562:ARG:O	1:A:563:ILE:HG13	1.78	0.83
1:A:229:LEU:HD21	1:A:291:LEU:CG	2.08	0.82
1:A:229:LEU:CD2	1:A:291:LEU:CG	2.57	0.82
1:A:364:ASN:HD22	1:A:364:ASN:H	1.28	0.81
1:A:452:GLU:HG3	1:A:482:LYS:HB2	1.63	0.81
1:A:626:LYS:HB2	1:A:627:PRO:CD	2.11	0.80
1:A:9:LEU:HD22	1:A:97:GLN:NE2	1.97	0.79
1:A:406:ILE:HD11	1:A:445:LEU:HD23	1.65	0.78
1:A:139:LEU:HD12	1:A:340:LEU:CA	2.15	0.77
1:A:670:THR:HG22	1:A:672:SER:H	1.50	0.76
1:A:139:LEU:HB2	1:A:340:LEU:HD13	1.67	0.76
1:A:260:TRP:HZ2	3:A:906:HOH:O	1.70	0.75
1:A:229:LEU:CD2	1:A:291:LEU:CD1	2.55	0.75
1:A:310:ASN:O	1:A:320:LEU:HD12	1.86	0.75
1:A:364:ASN:H	1:A:364:ASN:ND2	1.84	0.74
1:A:450:ASN:HA	1:A:482:LYS:HD3	1.69	0.74
1:A:430:TRP:HE1	1:A:435:THR:HB	1.50	0.74

Continued on next page...

CONFIDENTIAL VALIDATION REPORT

Continued from previous page...

Atom-1	Atom-2	Interatomic distance (Å)	Clash overlap (Å)
1:A:234:ASN:HD22	1:A:298:THR:CG2	2.01	0.73
1:A:229:LEU:HD23	1:A:291:LEU:HG	1.72	0.72
1:A:139:LEU:HD12	1:A:340:LEU:N	2.05	0.71
1:A:255:LEU:HB2	1:A:316:ASP:HB2	1.71	0.71
1:A:260:TRP:CZ2	3:A:906:HOH:O	2.44	0.70
1:A:676:ALA:O	1:A:680:ASN:ND2	2.23	0.70
1:A:246:ARG:CB	1:A:371:GLY:O	2.40	0.70
1:A:568:ASP:OD1	1:A:570:THR:HG22	1.92	0.70
1:A:229:LEU:CD2	1:A:291:LEU:HD21	2.22	0.69
1:A:697:LYS:N	1:A:698:PRO:HD3	2.07	0.69
1:A:229:LEU:CD2	1:A:291:LEU:HG	2.22	0.69
1:A:186:ASP:O	1:A:190:GLN:HG2	1.92	0.69
1:A:406:ILE:CD1	1:A:445:LEU:CD2	2.63	0.69
1:A:715:LYS:HD3	1:A:718:ILE:CD1	2.21	0.69
1:A:669:ILE:HG12	1:A:734:HIS:NE2	2.08	0.69
1:A:139:LEU:HD11	1:A:339:LEU:HB2	1.74	0.69
1:A:313:VAL:HG13	1:A:318:ILE:HG12	1.73	0.69
1:A:691:PHE:CZ	1:A:706:LYS:HG2	2.29	0.68
1:A:229:LEU:HD23	1:A:291:LEU:CG	2.25	0.66
1:A:699:LEU:O	1:A:702:ILE:HG13	1.94	0.66
1:A:39:LEU:HB3	1:A:62:TYR:CE2	2.31	0.66
1:A:297:PHE:CE2	1:A:315:VAL:O	2.48	0.66
1:A:301:VAL:HG11	1:A:313:VAL:HG11	1.77	0.66
1:A:432:GLU:O	1:A:435:THR:CG2	2.43	0.66
1:A:463:LYS:NZ	1:A:568:ASP:OD2	2.29	0.66
1:A:220:TYR:O	1:A:224:ASN:ND2	2.29	0.66
1:A:618:MSE:HE2	1:A:639:LEU:HD21	1.78	0.65
1:A:385:PRO:HG2	1:A:685:TYR:HE1	1.61	0.65
1:A:248:LYS:HB2	1:A:370:ARG:NH2	2.12	0.65
1:A:49:LEU:HD12	1:A:49:LEU:O	1.97	0.65
1:A:246:ARG:CZ	1:A:374:ASP:HA	2.28	0.64
1:A:618:MSE:SE	1:A:620:ILE:HD11	2.49	0.63
1:A:594:TYR:HD2	1:A:665:PRO:HB2	1.63	0.63
1:A:234:ASN:OD1	1:A:235:GLN:N	2.32	0.62
1:A:384:THR:HG23	1:A:385:PRO:HD2	1.81	0.62
1:A:420:ILE:HG13	1:A:421:ALA:N	2.14	0.62
1:A:385:PRO:HG2	1:A:685:TYR:CE1	2.34	0.62
1:A:583:SER:HB2	1:A:585:GLN:OE1	1.99	0.62
1:A:403:ARG:HG2	1:A:409:PHE:CE2	2.34	0.62
1:A:249:TYR:CE1	1:A:325:PRO:HG3	2.34	0.62
1:A:139:LEU:HD12	1:A:340:LEU:HB2	1.82	0.62

Continued on next page...

CONFIDENTIAL VALIDATION REPORT

Continued from previous page...

Atom-1	Atom-2	Interatomic distance (Å)	Clash overlap (Å)
1:A:245:VAL:HG21	1:A:310:ASN:OD1	2.00	0.62
1:A:244:ILE:HD13	1:A:367:LYS:NZ	2.15	0.61
1:A:437:GLN:O	1:A:441:VAL:HG23	2.00	0.61
1:A:676:ALA:CB	1:A:722:HIS:HB3	2.31	0.61
1:A:417:TYR:O	1:A:420:ILE:HG12	2.01	0.61
1:A:426:LYS:C	3:A:903:HOH:O	2.39	0.61
1:A:49:LEU:HA	1:A:58:GLN:HA	1.83	0.60
1:A:666:VAL:CG2	1:A:718:ILE:HG12	2.32	0.60
1:A:109:GLN:O	1:A:113:GLU:HG3	2.03	0.59
1:A:669:ILE:HG12	1:A:734:HIS:CE1	2.36	0.59
1:A:246:ARG:NH1	1:A:374:ASP:HA	2.17	0.59
1:A:408:ASN:O	1:A:412:LEU:HD23	2.03	0.59
1:A:229:LEU:CD2	1:A:291:LEU:CD2	2.81	0.59
1:A:139:LEU:CD1	1:A:340:LEU:N	2.65	0.58
1:A:21:LEU:HD11	1:A:83:GLU:O	2.04	0.58
1:A:137:ASP:CA	1:A:140:TRP:CD1	2.80	0.58
1:A:25:TRP:CZ2	1:A:89:PRO:HB3	2.38	0.58
1:A:229:LEU:HD23	1:A:291:LEU:CD2	2.34	0.58
1:A:649:ILE:HG12	1:A:675:VAL:HG22	1.86	0.57
1:A:39:LEU:HB3	1:A:62:TYR:HE2	1.69	0.57
1:A:494:ALA:HA	1:A:497:LYS:HE3	1.87	0.57
1:A:348:LEU:HA	1:A:351:SER:HB2	1.87	0.57
1:A:405:PHE:CE2	1:A:406:ILE:HG13	2.40	0.57
1:A:553:THR:O	1:A:556:THR:HG22	2.05	0.57
1:A:259:PHE:O	1:A:286:LEU:HB2	2.05	0.57
1:A:14:ALA:HB1	1:A:67:LYS:O	2.04	0.57
1:A:727:GLN:N	1:A:728:PRO:HD3	2.19	0.57
1:A:139:LEU:HD12	1:A:340:LEU:CB	2.35	0.56
1:A:244:ILE:O	1:A:244:ILE:HG13	2.05	0.56
1:A:416:LEU:O	1:A:419:LEU:HB2	2.05	0.56
1:A:34:ILE:HD12	1:A:77:VAL:HG21	1.86	0.56
1:A:324:ASP:OD1	1:A:326:LYS:HG2	2.05	0.56
1:A:134:VAL:HG12	1:A:134:VAL:O	2.05	0.56
1:A:229:LEU:HG	1:A:291:LEU:HD21	1.87	0.56
1:A:643:THR:HG22	1:A:645:GLN:H	1.71	0.56
1:A:618:MSE:CE	1:A:639:LEU:HD21	2.36	0.55
1:A:697:LYS:N	1:A:698:PRO:HD2	2.22	0.55
1:A:259:PHE:CE1	1:A:288:GLN:HB3	2.41	0.55
1:A:229:LEU:HD23	1:A:291:LEU:HD21	1.87	0.55
1:A:150:ARG:HD2	1:A:342:ALA:O	2.07	0.55
1:A:585:GLN:HG2	1:A:680:ASN:HB3	1.88	0.55

Continued on next page...

CONFIDENTIAL VALIDATION REPORT

Continued from previous page...

Atom-1	Atom-2	Interatomic distance (Å)	Clash overlap (Å)
1:A:674:TYR:HE2	1:A:705:TRP:HZ2	1.55	0.55
1:A:415:PRO:O	1:A:419:LEU:HD23	2.06	0.55
1:A:402:ALA:CB	1:A:406:ILE:HD12	2.20	0.54
1:A:562:ARG:C	1:A:563:ILE:HG13	2.28	0.54
1:A:155:HIS:O	1:A:265:THR:N	2.40	0.54
1:A:164:PRO:HD3	1:A:226:THR:HG21	1.91	0.54
1:A:198:SER:OG	1:A:270:TRP:HB2	2.06	0.54
1:A:585:GLN:CG	1:A:680:ASN:HB3	2.39	0.53
1:A:364:ASN:HB3	1:A:550:ARG:HH12	1.72	0.53
1:A:641:ASN:O	1:A:642:HIS:HD2	1.91	0.53
1:A:169:GLN:OE1	1:A:204:VAL:HG12	2.08	0.53
1:A:384:THR:HG21	1:A:688:SER:HB2	1.90	0.53
1:A:22:LEU:HD11	1:A:191:GLY:HA3	1.91	0.52
1:A:618:MSE:SE	1:A:651:ALA:HA	2.59	0.52
1:A:246:ARG:CG	1:A:371:GLY:O	2.56	0.52
1:A:417:TYR:C	1:A:419:LEU:H	2.12	0.52
1:A:16:ILE:HG12	1:A:17:LYS:HG3	1.90	0.52
1:A:471:GLY:H	1:A:489:TYR:HB3	1.73	0.52
1:A:402:ALA:HA	1:A:405:PHE:CE1	2.45	0.52
1:A:666:VAL:CG2	1:A:718:ILE:HG23	2.33	0.52
1:A:626:LYS:HB3	1:A:627:PRO:CD	2.26	0.52
1:A:16:ILE:HG23	1:A:17:LYS:H	1.74	0.52
1:A:234:ASN:HD22	1:A:298:THR:HG22	1.75	0.52
1:A:380:LEU:HB2	1:A:488:ASN:HB2	1.90	0.52
1:A:447:THR:HA	3:A:908:HOH:O	2.10	0.51
1:A:528:SER:OG	1:A:530:ILE:HG12	2.10	0.51
1:A:452:GLU:OE1	1:A:482:LYS:N	2.37	0.51
1:A:380:LEU:HD12	1:A:380:LEU:C	2.31	0.51
1:A:222:GLU:O	1:A:225:LYS:HG2	2.10	0.51
1:A:641:ASN:C	1:A:642:HIS:HD2	2.15	0.51
1:A:604:ALA:HB3	1:A:644:ALA:HB2	1.92	0.51
1:A:25:TRP:CE3	1:A:97:GLN:HG3	2.47	0.50
1:A:691:PHE:CE2	1:A:706:LYS:HG2	2.46	0.50
1:A:246:ARG:HG3	1:A:371:GLY:O	2.11	0.50
1:A:229:LEU:CG	1:A:291:LEU:HD21	2.42	0.50
1:A:300:ASP:OD1	1:A:304:LEU:HD13	2.11	0.50
1:A:33:CYS:HA	1:A:78:ILE:O	2.12	0.49
1:A:145:ASN:O	1:A:190:GLN:NE2	2.45	0.49
1:A:252:THR:HA	1:A:318:ILE:O	2.12	0.49
1:A:139:LEU:O	1:A:139:LEU:CD2	2.48	0.49
1:A:33:CYS:HB3	1:A:80:SER:HB2	1.94	0.49

Continued on next page...

CONFIDENTIAL VALIDATION REPORT

Continued from previous page...

Atom-1	Atom-2	Interatomic distance (Å)	Clash overlap (Å)
1:A:244:ILE:HD13	1:A:367:LYS:HZ3	1.76	0.49
1:A:240:ILE:O	1:A:244:ILE:HG23	2.12	0.49
1:A:25:TRP:CD2	1:A:97:GLN:HB2	2.47	0.49
1:A:45:ILE:HD11	1:A:63:TYR:HB2	1.95	0.49
1:A:173:ASN:OD1	1:A:174:PRO:HD2	2.12	0.49
1:A:228:PRO:HB2	1:A:230:THR:HG23	1.94	0.49
1:A:618:MSE:HB3	1:A:639:LEU:HD11	1.94	0.49
1:A:381:LEU:HD13	1:A:582:THR:HA	1.93	0.49
1:A:673:PHE:CD2	1:A:674:TYR:N	2.81	0.48
1:A:25:TRP:CE2	1:A:97:GLN:HB2	2.48	0.48
1:A:405:PHE:CD2	1:A:482:LYS:HG2	2.48	0.48
1:A:380:LEU:CB	1:A:488:ASN:HB2	2.43	0.48
1:A:103:THR:HG22	1:A:104:ILE:HG13	1.96	0.48
1:A:139:LEU:HD11	1:A:339:LEU:CB	2.42	0.48
1:A:394:SER:OG	1:A:576:HIS:HB2	2.14	0.48
1:A:123:GLU:HA	1:A:126:LYS:HB3	1.95	0.48
1:A:359:GLU:O	1:A:366:THR:HG22	2.14	0.48
1:A:150:ARG:HH21	1:A:304:LEU:HD11	1.77	0.47
1:A:132:LEU:HD21	1:A:333:GLU:OE2	2.14	0.47
1:A:95:LEU:HD12	1:A:96:THR:H	1.79	0.47
1:A:249:TYR:HE1	1:A:325:PRO:HG3	1.78	0.47
1:A:691:PHE:CD2	1:A:706:LYS:HE2	2.49	0.47
1:A:87:LEU:HD21	1:A:92:VAL:HG21	1.97	0.47
1:A:108:LEU:O	1:A:112:GLN:HG3	2.15	0.47
1:A:252:THR:HG22	1:A:352:GLU:HB2	1.95	0.47
1:A:674:TYR:O	1:A:678:SER:HB2	2.14	0.47
1:A:118:LYS:O	1:A:355:GLN:NE2	2.45	0.47
1:A:682:GLU:HB3	1:A:686:TRP:CD1	2.49	0.47
1:A:250:LYS:HB3	1:A:319:TYR:HE1	1.80	0.47
1:A:562:ARG:O	1:A:563:ILE:CG1	2.57	0.47
1:A:600:THR:OG1	1:A:669:ILE:O	2.22	0.47
1:A:378:THR:O	1:A:486:TYR:O	2.32	0.46
1:A:420:ILE:HG13	1:A:421:ALA:H	1.80	0.46
1:A:624:THR:HG23	1:A:632:LEU:HG	1.97	0.46
1:A:641:ASN:O	1:A:642:HIS:CD2	2.67	0.46
1:A:726:HIS:C	1:A:728:PRO:HD3	2.36	0.46
1:A:152:ILE:HG12	1:A:153:ARG:H	1.81	0.46
1:A:35:PRO:HA	1:A:80:SER:O	2.15	0.46
1:A:483:PRO:HG2	1:A:486:TYR:CZ	2.51	0.46
1:A:267:GLU:HG2	1:A:267:GLU:O	2.15	0.46
1:A:725:GLY:C	1:A:728:PRO:HG3	2.36	0.46

Continued on next page...

CONFIDENTIAL VALIDATION REPORT

Continued from previous page...

Atom-1	Atom-2	Interatomic distance (Å)	Clash overlap (Å)
1:A:508:MSE:HE1	1:A:530:ILE:HD11	1.96	0.46
1:A:424:LYS:HG2	1:A:424:LYS:O	2.16	0.45
1:A:445:LEU:HD22	1:A:486:TYR:OH	2.17	0.45
1:A:177:LYS:HA	1:A:276:TRP:HH2	1.82	0.45
1:A:378:THR:HG23	1:A:485:MSE:HE3	1.99	0.45
1:A:137:ASP:HA	1:A:140:TRP:NE1	2.27	0.45
1:A:44:PRO:HA	1:A:62:TYR:CD1	2.52	0.45
1:A:509:ASP:O	1:A:513:ILE:HG13	2.17	0.44
1:A:192:VAL:HG13	1:A:285:ARG:HG3	2.00	0.44
1:A:21:LEU:HD12	1:A:22:LEU:H	1.83	0.44
1:A:660:LEU:HD21	1:A:711:CYS:SG	2.57	0.44
1:A:729:THR:OG1	1:A:730:ASN:N	2.50	0.44
1:A:255:LEU:HD11	1:A:318:ILE:HD11	1.98	0.44
1:A:381:LEU:HB2	1:A:580:VAL:CG1	2.48	0.44
1:A:541:LEU:HA	1:A:544:ARG:HG3	1.99	0.44
1:A:503:LYS:HA	1:A:506:THR:HG22	1.98	0.44
1:A:115:ILE:O	1:A:119:THR:OG1	2.32	0.44
1:A:187:LEU:HD23	1:A:190:GLN:HE21	1.82	0.44
1:A:255:LEU:O	1:A:316:ASP:OD2	2.36	0.44
1:A:181:GLN:OE1	1:A:276:TRP:NE1	2.43	0.44
1:A:646:MSE:HG2	1:A:646:MSE:O	2.16	0.44
1:A:666:VAL:HG22	1:A:718:ILE:HG12	1.99	0.44
1:A:676:ALA:HB3	1:A:722:HIS:HB3	2.00	0.44
1:A:537:GLN:OE1	1:A:555:MSE:HE2	2.18	0.44
1:A:502:GLU:OE2	1:A:544:ARG:HA	2.18	0.43
1:A:716:PRO:C	1:A:718:ILE:H	2.22	0.43
1:A:364:ASN:HD22	1:A:364:ASN:N	2.07	0.43
1:A:467:SER:HB2	1:A:577:ILE:HB	2.00	0.43
1:A:374:ASP:N	1:A:374:ASP:OD1	2.52	0.43
1:A:139:LEU:HD12	1:A:340:LEU:HA	1.99	0.43
1:A:153:ARG:HA	1:A:154:PRO:HD3	1.92	0.43
1:A:405:PHE:CD2	1:A:406:ILE:HG13	2.54	0.43
1:A:32:THR:HG23	1:A:87:LEU:HA	2.00	0.43
1:A:8:LEU:HD11	1:A:138:ASN:HB3	2.01	0.43
1:A:244:ILE:HG22	1:A:312:GLN:HG3	2.00	0.43
1:A:237:SER:O	1:A:241:LEU:HG	2.19	0.42
1:A:246:ARG:HE	1:A:371:GLY:H	1.67	0.42
1:A:305:LEU:HD13	1:A:311:VAL:HG21	2.00	0.42
1:A:132:LEU:HD23	1:A:132:LEU:HA	1.76	0.42
1:A:152:ILE:HG12	1:A:153:ARG:N	2.34	0.42
1:A:389:LEU:HB2	1:A:426:LYS:O	2.20	0.42

Continued on next page...

CONFIDENTIAL VALIDATION REPORT

Continued from previous page...

Atom-1	Atom-2	Interatomic distance (Å)	Clash overlap (Å)
1:A:198:SER:OG	1:A:199:THR:N	2.53	0.42
1:A:519:ALA:O	1:A:522:GLN:HB2	2.19	0.42
1:A:551:TRP:O	1:A:555:MSE:HG2	2.19	0.42
1:A:641:ASN:C	1:A:642:HIS:CD2	2.93	0.42
1:A:530:ILE:HB	1:A:536:ILE:HD11	2.02	0.42
1:A:224:ASN:OD1	1:A:291:LEU:N	2.53	0.42
1:A:518:LEU:O	1:A:520:MSE:HG3	2.20	0.42
1:A:594:TYR:CD2	1:A:665:PRO:HB2	2.48	0.42
1:A:642:HIS:ND1	1:A:646:MSE:SE	3.03	0.42
1:A:476:TYR:CZ	1:A:483:PRO:HG3	2.55	0.41
1:A:683:LEU:HB3	1:A:684:PRO:HD3	2.03	0.41
1:A:359:GLU:O	1:A:360:PHE:CG	2.74	0.41
1:A:622:HIS:HB3	1:A:633:ASN:H	1.85	0.41
1:A:674:TYR:CE2	1:A:705:TRP:HZ2	2.38	0.41
1:A:247:GLN:OE1	1:A:322:HIS:HA	2.21	0.41
1:A:645:GLN:O	1:A:649:ILE:HG13	2.20	0.41
1:A:599:TYR:CZ	1:A:737:GLY:HA3	2.56	0.41
1:A:116:LEU:CD2	1:A:129:LEU:HD23	2.51	0.41
1:A:11:PRO:O	1:A:12:LEU:HD23	2.21	0.41
1:A:442:ILE:HG22	1:A:446:ASN:ND2	2.35	0.41
1:A:240:ILE:HG23	1:A:312:GLN:OE1	2.21	0.41
1:A:314:TYR:O	1:A:316:ASP:N	2.54	0.41
1:A:234:ASN:HD22	1:A:298:THR:HG21	1.81	0.41
1:A:704:LYS:HA	1:A:704:LYS:HD3	1.88	0.41
1:A:431:SER:N	1:A:434:ASN:OD1	2.46	0.41
1:A:595:GLU:HG2	1:A:595:GLU:O	2.20	0.41
1:A:293:SER:HB2	1:A:294:PRO:HD3	2.03	0.40
1:A:684:PRO:HA	1:A:687:LYS:HD2	2.03	0.40
1:A:174:PRO:CA	1:A:177:LYS:HG3	2.33	0.40
1:A:25:TRP:CE2	1:A:89:PRO:HB3	2.55	0.40
1:A:219:ASP:OD1	1:A:221:ARG:HG2	2.21	0.40
1:A:301:VAL:HG12	1:A:313:VAL:HG21	2.04	0.40
1:A:417:TYR:C	1:A:419:LEU:N	2.73	0.40
1:A:697:LYS:N	1:A:701:HIS:HB3	2.35	0.40
1:A:106:VAL:HG22	1:A:142:HIS:O	2.21	0.40
1:A:251:THR:O	1:A:319:TYR:HA	2.22	0.40
1:A:598:PHE:CG	1:A:655:ALA:HB1	2.57	0.40
1:A:743:LYS:O	1:A:747:GLN:HG3	2.22	0.40

There are no symmetry-related clashes.

CONFIDENTIAL VALIDATION REPORT

5.3 Torsion angles (i)

5.3.1 Protein backbone (i)

In the following table, the Percentiles column shows the percent Ramachandran outliers of the chain as a percentile score with respect to all X-ray entries followed by that with respect to entries of similar resolution.

The Analysed column shows the number of residues for which the backbone conformation was analysed, and the total number of residues.

Mol	Chain	Analysed	Favoured	Allowed	Outliers	Percentiles
1	A	736/747 (98%)	655 (89%)	76 (10%)	5 (1%)	22 60

All (5) Ramachandran outliers are listed below:

Mol	Chain	Res	Type
1	A	626	LYS
1	A	315	VAL
1	A	426	LYS
1	A	699	LEU
1	A	428	ILE

5.3.2 Protein sidechains (i)

In the following table, the Percentiles column shows the percent sidechain outliers of the chain as a percentile score with respect to all X-ray entries followed by that with respect to entries of similar resolution.

The Analysed column shows the number of residues for which the sidechain conformation was analysed, and the total number of residues.

Mol	Chain	Analysed	Rotameric	Outliers	Percentiles
1	A	648/656 (99%)	645 (100%)	3 (0%)	88 96

All (3) residues with a non-rotameric sidechain are listed below:

Mol	Chain	Res	Type
1	A	215	ARG
1	A	259	PHE
1	A	364	ASN

Sometimes sidechains can be flipped to improve hydrogen bonding and reduce clashes. All (3) such sidechains are listed below:

CONFIDENTIAL VALIDATION REPORT

Mol	Chain	Res	Type
1	A	97	GLN
1	A	364	ASN
1	A	446	ASN

5.3.3 RNA (i)

There are no RNA molecules in this entry.

5.4 Non-standard residues in protein, DNA, RNA chains (i)

There are no non-standard protein/DNA/RNA residues in this entry.

5.5 Carbohydrates (i)

There are no monosaccharides in this entry.

5.6 Ligand geometry (i)

Of 1 ligands modelled in this entry, 1 is monoatomic - leaving 0 for Mogul analysis.

There are no bond length outliers.

There are no bond angle outliers.

There are no chirality outliers.

There are no torsion outliers.

There are no ring outliers.

No monomer is involved in short contacts.

5.7 Other polymers (i)

There are no such residues in this entry.

5.8 Polymer linkage issues (i)

There are no chain breaks in this entry.

CONFIDENTIAL VALIDATION REPORT

6 Fit of model and data (i)

6.1 Protein, DNA and RNA chains (i)

In the following table, the column labelled '#RSRZ> 2' contains the number (and percentage) of RSRZ outliers, followed by percent RSRZ outliers for the chain as percentile scores relative to all X-ray entries and entries of similar resolution. The OWAB column contains the minimum, median, 95th percentile and maximum values of the occupancy-weighted average B-factor per residue. The column labelled 'Q< 0.9' lists the number of (and percentage) of residues with an average occupancy less than 0.9.

Mol	Chain	Analysed	<RSRZ>	#RSRZ>2			OWAB(Å^2)	Q<0.9
1	A	729/747 (97%)	0.28	34 (4%)	31	11	41, 83, 139, 183	1 (0%)

All (34) RSRZ outliers are listed below:

Mol	Chain	Res	Type	RSRZ
1	A	364	ASN	7.9
1	A	694	ASN	6.9
1	A	700	LYS	6.4
1	A	232	ALA	5.4
1	A	698	PRO	5.3
1	A	52	THR	4.9
1	A	234	ASN	4.7
1	A	362	GLY	4.7
1	A	721	GLN	4.1
1	A	726	HIS	4.1
1	A	235	GLN	3.9
1	A	376	PHE	3.8
1	A	457	GLU	3.3
1	A	678	SER	3.1
1	A	699	LEU	3.0
1	A	588	VAL	2.9
1	A	427	TYR	2.8
1	A	479	THR	2.8
1	A	703	SER	2.7
1	A	681	LYS	2.7
1	A	586	LYS	2.6
1	A	675	VAL	2.6
1	A	233	GLN	2.5
1	A	230	THR	2.5
1	A	724	LYS	2.5
1	A	422	SER	2.4
1	A	391	GLN	2.4

Continued on next page...

CONFIDENTIAL VALIDATION REPORT

Continued from previous page...

Mol	Chain	Res	Type	RSRZ
1	A	686	TRP	2.3
1	A	587	PRO	2.2
1	A	394	SER	2.2
1	A	373	THR	2.1
1	A	729	THR	2.1
1	A	591	PRO	2.1
1	A	691	PHE	2.1

6.2 Non-standard residues in protein, DNA, RNA chains (i)

There are no non-standard protein/DNA/RNA residues in this entry.

6.3 Carbohydrates (i)

There are no monosaccharides in this entry.

6.4 Ligands (i)

In the following table, the Atoms column lists the number of modelled atoms in the group and the number defined in the chemical component dictionary. The B-factors column lists the minimum, median, 95th percentile and maximum values of B factors of atoms in the group. The column labelled 'Q< 0.9' lists the number of atoms with occupancy less than 0.9.

Mol	Type	Chain	Res	Atoms	RSCC	RSR	B-factors(Å^2)	Q<0.9
2	CA	A	801	1/1	0.78	0.50	113,113,113,113	0

6.5 Other polymers (i)

There are no such residues in this entry.

CONFIDENTIAL VALIDATION REPORT
