# Supplementary material for: Crystal Structure of a Retroviral Polyprotein: Prototype Foamy Virus Protease-Reverse Transcriptase (PR-RT)
Source: Viruses. 2021 Jul 29;13(8):1495. doi: 10.3390/v13081495 (PMC8402755; doi:10.3390/v13081495)
Supplement: Supplementary file 1 [file viruses-13-01495-s001.zip › supplementary material.pdf]

SUPPLEMENTAL MATERIAL

Crystal Structure of a Retroviral Polyprotein: Prototype Foamy Virus Protease-Reverse Transcriptase (PR-RT)

Jerry Joe E. K. Harrison [1,2,3], Steve Tuske [1], Kalyan Das [1,4], Francesc X. Ruiz [1], Joseph D. Bauman [1], Paul L. Boyer [5], Jeffrey J. DeStefano [6], Stephen H. Hughes [5] and Eddy Arnold [1,2,7,*]

[1] Center for Advanced Biotechnology and Medicine (CABM), Rutgers University, Piscataway, NJ 08854, USA; jjharrison@ug.edu.gh (J.J.E.K.H.); s_tuske@hotmail.com (S.T.); kalyan.das@kuleuven.be (K.D.); xavier@cabm.rutgers.edu (F.X.R.); marinesci@me.com (J.D.B.)

[2] Department of Medicinal Chemistry, Ernest Mario School of Pharmacy, Rutgers University, Piscataway, NJ 08854, USA

[3] Department of Chemistry, University of Ghana, Legon P.O. Box LG 56, Ghana

[4] Department of Microbiology, Immunology and Transplantation, Rega Institute, KU Leuven, 3000 Leuven, Belgium

[5] HIV Dynamics and Replication Program, National Cancer Institute, Frederick, MD 21702, USA; boyerp@mail.nih.gov (P.L.B.); hughesst@mail.nih.gov (S.H.H.)

[6] Department of Cell Biology and Molecular Genetics, University of Maryland College Park, College Park, MD 20742, USA; jdestefa@umd.edu

[7] Department of Chemistry and Chemical Biology, Rutgers University, Piscataway, NJ 08854, USA

* Correspondence: arnold@cabm.rutgers.edu

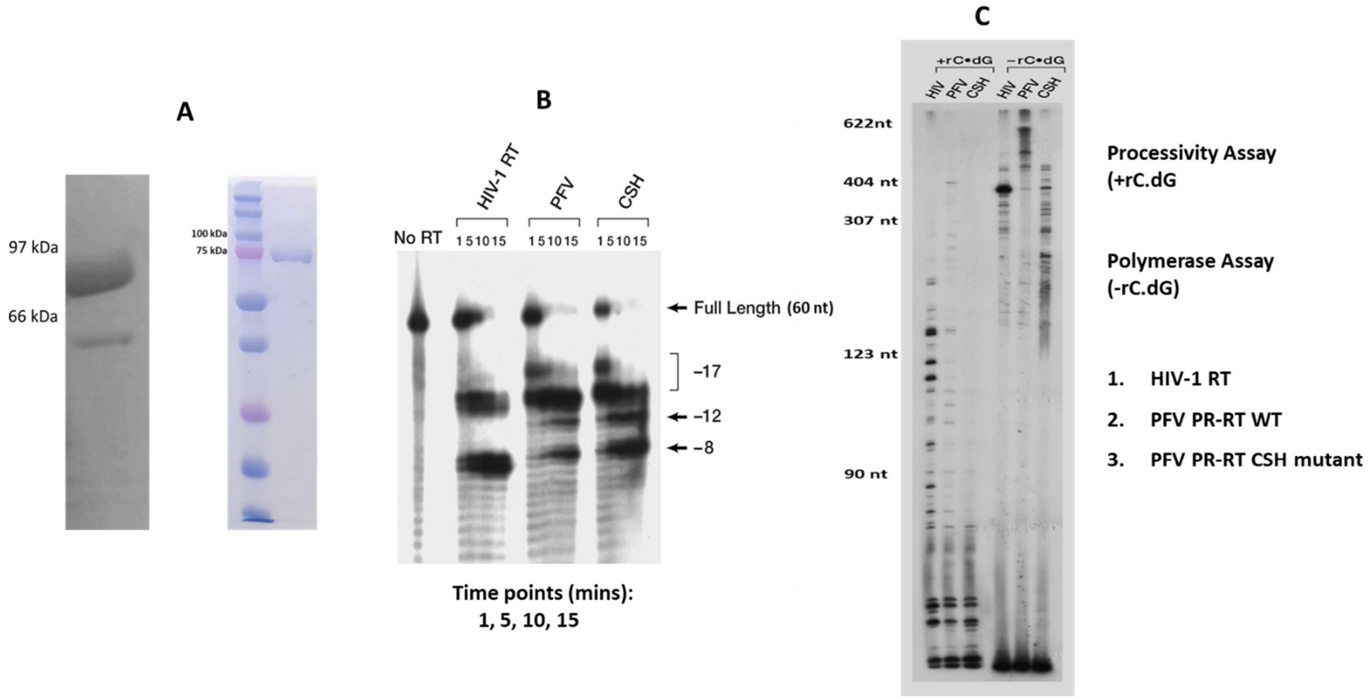

Figure S1. (A) SDS-PAGE gel of PFV PR-RT WT (left) and the CSH mutant (right) after purification. (B) Ribonuclease H activity of PR-RT and HIV-1 RT. (C) Polymerase activity and processivity (as in reference 30 of the main manuscript, Boyer et al., 2004, PMID: 15163704). The polymerase assays analyzed in three lanes on the right were done with a labeled primer annealed to single-stranded M13mp18 DNA. A polyrC/oligodG unlabeled trap, which limits DNA synthesis to a single round of PR-RT binding, was used in the three lanes on the left (+rC.dG), the assays were done without a trap, and the PR-RT can rebind if it dissociates from the substrate.

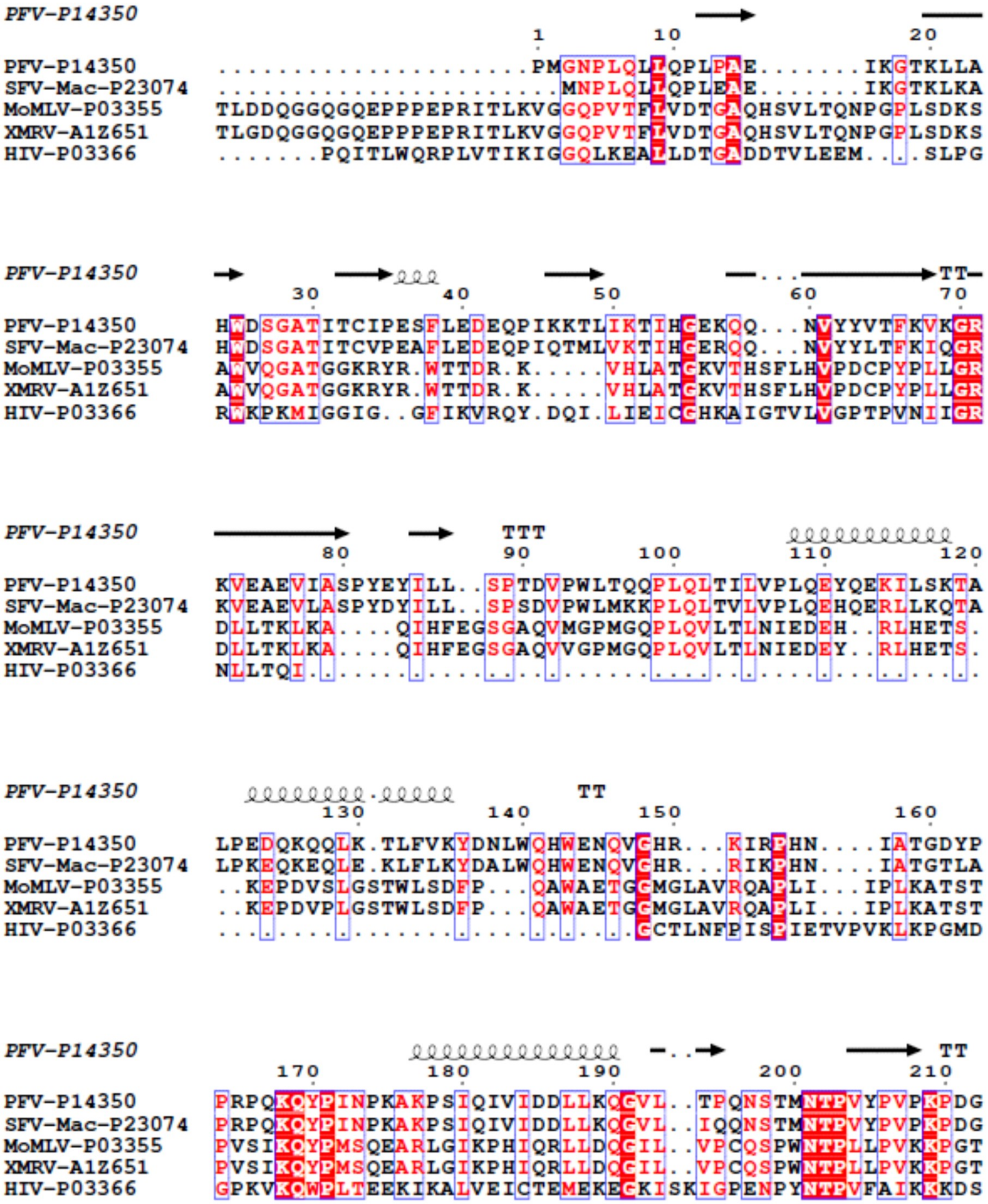
PFV-P14350
1 10 20
PFV-P14350 .....................PMGNPLQLIQPLPAE.......IKGTKLLA
SFV-Mac-P23074 .......................MNPLQLIQPLEAE.......IKGTKLKA
MoMLV-P03355 TLDDQGGQGQEPPPEPRITLKVGGQPVTFLVDTGAQHSVLTQNPGPLSDKS
XMRV-A1Z651 TLGDQGGQGQEPPPEPRITLKVGGQPVTFLVDTGAQHSVLTQNPGPLSDKS
HIV-P03366 .......PQITLWQRPLVTIKIGGQLKEALLDTGADDTVLEEM....SLPG
PFV-P14350
TT
30 40 50 60 70
PFV-P14350 HWDSGATITCIPESFLEDEQPIKKTLIKTIHGEKQQ...NVYYVTFKVKGR
SFV-Mac-P23074 HWDSGATITCVPEAFLEDEQPIQTMLVKTIHGERQQ...NVYYLTFKIQGR
MoMLV-P03355 AWVQGATGGKRYR.WTTDR.K.....VHLATGKVTHSFLHVPDCPYPLLGR
XMRV-A1Z651 AWVQGATGGKRYR.WTTDR.K.....VHLATGKVTHSFLHVPDCPYPLLGR
HIV-P03366 RWKPKMIGGIG..GFIKVRQY.DQI.LIEICGHKAIGTVLVGPTPVNIIGR
PFV-P14350
TTT
80 90 100 110 120
PFV-P14350 KVEAEVIASPYEYILL..SPTDVPWLTQQPLQLTILVPLQEYQEKILSKTA
SFV-Mac-P23074 KVEAEVLASPYDYILL..SPSDVPWLMKKPLQLTVLVPLQEHQERLLKQTA
MoMLV-P03355 DLLTKLKA....QIHFEGSGAQVMGPMGQPLQVLTLNIEDEH..RLHETS.
XMRV-A1Z651 DLLTKLKA....QIHFEGSGAQVVGPMGQPLQVLTLNIEDEY..RLHETS.
HIV-P03366 NLLTQI...
PFV-P14350
TT
130 140 150 160
PFV-P14350 LPEDQKQQLK.TLFVKYDNLWQHWENQVGHR...KIRPHN....IATGDYP
SFV-Mac-P23074 LPKEQKEQLE.KLFLKYDALWQHWENQVGHR...RIKPHN....IATGTLA
MoMLV-P03355 ..KEPDVSLGSTWLSDFP...QAWAETGGMGLAVRQAPLI...IPLKATST
XMRV-A1Z651 ..KEPDVPLGSTWLSDFP...QAWAETGGMGLAVRQAPLI...IPLKATST
HIV-P03366 ............................GCTLNFPISPIETVPVKLKPGMD
PFV-P14350
TT
170 180 190 200 210
PFV-P14350 PRPQKQYPINPKAKPSIQIVIDDLLKQGVL..TPQNSTMNTPVYPVPKPDG
SFV-Mac-P23074 PRPQKQYPINPKAKPSIQIVIDDLLKQGVL..IQQNSTMNTPVYPVPKPDG
MoMLV-P03355 PVSIKQYPMSQEARLGIKPHIQRLLDQGIL..VPCQSPWNTPLLPVKKPGT
XMRV-A1Z651 PVSIKQYPMSQEARLGIKPHIQRLLDQGIL..VPCQSPWNTPLLPVKKPGT
HIV-P03366 GPKVKQWPLTEEKIKALVEICTEMEKEGKISKIGPENPYNTPVFAIKKKDS

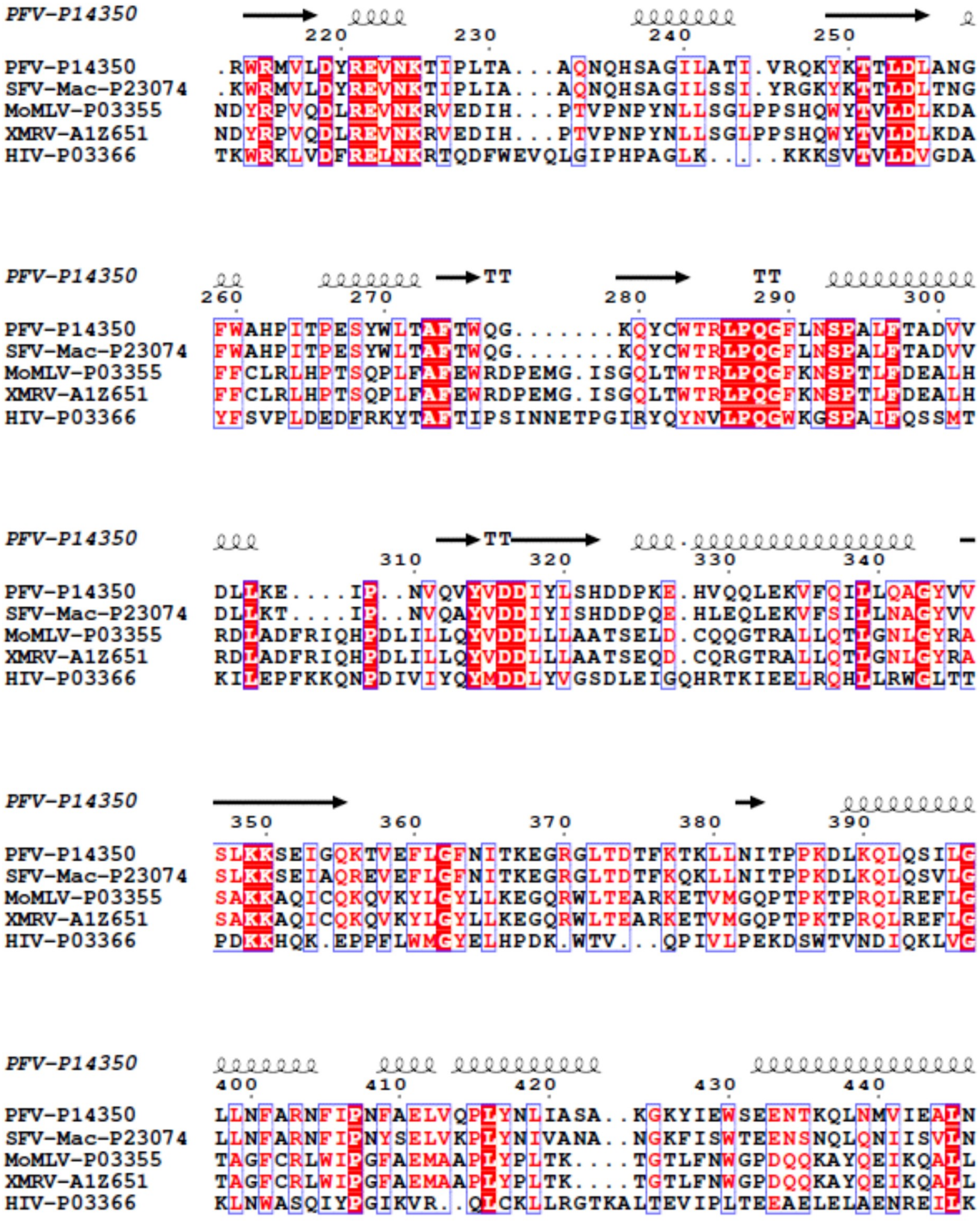
PFV-P14350
220 230 240 250
PFV-P14350 .RWRMVLDYREVNKTIPLTA...AQNQHSAGILATI.VRQKYKTTLDLANG
SFV-Mac-P23074 .KWRMVLDYREVNKTIPLIA...AQNQHSAGILSSI.YRGKYKTTLDLTNG
MoMLV-P03355 NDYRPVQDLREVNKRVEDIH...PTVPNPYNLLSGLPPSHQWYTVLDLKDA
XMRV-A1Z651 NDYRPVQDLREVNKRVEDIH...PTVPNPYNLLSGLPPSHQWYTVLDLKDA
HIV-P03366 TKWRKLVDFRELNKRTQDFWEVQLGIPHPAGLK.....KKKSVTVLDVGDA
PFV-P14350
TT TT
260 270 280 290 300
PFV-P14350 FWAHPITPESYWLTAFTWQG.......KQYCWTRLPQGFLNSPALFTADVV
SFV-Mac-P23074 FWAHPITPESYWLTAFTWQG.......KQYCWTRLPQGFLNSPALFTADVV
MoMLV-P03355 FFCLRLHPTSQPLFAFEWRDPEMG.ISGQLTWTRLPQGFKNSPTLFDEALH
XMRV-A1Z651 FFCLRLHPTSQPLFAFEWRDPEMG.ISGQLTWTRLPQGFKNSPTLFDEALH
HIV-P03366 YFSVPLDEDFRKYTAFTIPSINNETPGIRYQYNVLPQGWKGSPAIFQSSMT
PFV-P14350
TT
310 320 330 340
PFV-P14350 DLLKE....IP..NVQVYVDDIYLSHDDPKE.HVQQLEKVFQILLQAGYVV
SFV-Mac-P23074 DLLKT....IP..NVQAYVDDIYISHDDPQE.HLEQLEKVFSILLNAGYVV
MoMLV-P03355 RDLADFRIQHPDLILLQYVDDLLLAATSELD.CQQGTRALLQTLGNLGYRA
XMRV-A1Z651 RDLADFRIQHPDLILLQYVDDLLLAATSEQD.CQRGTRALLQTLGNLGYRA
HIV-P03366 KILEPFKKQNPDIVIYQYMDDLYVGSDLEIGQHRTKIEELRQHLLRWGLTT
PFV-P14350
350 360 370 380 390
PFV-P14350 SLKKSEIGQKTVEFLGFNITKEGRGLTDTFKTKLLNITPPKDLKQLQSILG
SFV-Mac-P23074 SLKKSEIAQREVEFLGFNITKEGRGLTDTFKQKLLNITPPKDLKQLQSVLG
MoMLV-P03355 SAKKAQICQKQVKYLGYLLKEGQRWLTEARKETVMGQPTPKTPRQLREFLG
XMRV-A1Z651 SAKKAQICQKQVKYLGYLLKEGQRWLTEARKETVMGQPTPKTPRQLREFLG
HIV-P03366 PDKKHQK.EPPFLWMGYELHPDK.WTV...QPIVLPEKDSWTVNDIQKLVG
PFV-P14350
400 410 420 430 440
PFV-P14350 LLNFARNFIPNFAELVQPLYNLIASA..KGKYIEWSEENTKQLNMVIEALN
SFV-Mac-P23074 LLNFARNFIPNYSELVKPLYNIVANA..NGKFISWTEENSNQLQNIISVLN
MoMLV-P03355 TAGFCRLWIPGFAEMAAPLYPLTK....TGTLFNWGPDQQKAYQEIKQALL
XMRV-A1Z651 TAGFCRLWIPGFAEMAAPLYPLTK....TGTLFNWGPDQQKAYQEIKQALL
HIV-P03366 KLNWASQIYPGIKVR..QLCKLLRGTKALTEVIPLTEEAELELAENREILK

```
PFV-P14350
                 450       460       470       480       490
PFV-P14350      TASNLEERLPEQ..RLVIKVNTSPSAGYV....RYYNETGKKPIMYLNYVF
SFV-Mac-P23074  QADNLEERNPET..RLIIKVNSSPSAGYI....RYYNEGSKRPIMYINYVF
MoMLV-P03355    TAPALGLPDLTKPFELFVDEKQGYAKGVL....TQKLGPWRRPVAYLSKKL
XMRV-A1Z651     TAPALGLPDLTKPFELFVDEKQGYAKGVL....TQKLGPWRRPVAYLSKKL
HIV-P03366      ......EP........VHGVYYDPSKDLIAEIQKQGQGQWTYQIYQE..PF
```

```
PFV-P14350
                     500       510       520       530       540
PFV-P14350      SKAELKFSMLEKLLTTMHKALIKAMDLAMGQEILVYSPIVSMTKIQKTPLP
SFV-Mac-P23074  SKAESKFTQTEKMLTTMHKGLIKAMDLAMGQEILVYSPIVSMTKIQKTPLP
MoMLV-P03355    DPVAAGWPPCLRMVAAIAVLTKDAGKLTMGQPLVILAPHAVEALV......
XMRV-A1Z651     DPVAAGWPPCLRMVAAIAVLTKDAGKLTMGQPLVILAPHAVEALV......
HIV-P03366      KN..LKTGKYARMRGAHT...NDVKQLTEAVQKITTESIVIWGKTPKFKLP
```

```
PFV-P14350                                    TT
                    550       560       570       580
PFV-P14350      ERKALPIRWITWMT.......YLEDPRIQFHYDKTLPELKHIPDVYTSSQ.
SFV-Mac-P23074  ERKALPVRWITWMT.......YLEDPRIQFHYDKTLPELQQIPSVTEDIV.
MoMLV-P03355    ..KQPPDRWLSNARMTHYQALLLDTDRVQFGPVVALNPATLLPLPEEGLQH
XMRV-A1Z651     ..KQPPDRWLSNARMTHYQAMLLDTDRVQFGPVVALNPATLLPLPEKEAPH
HIV-P03366      IQ...KETWETWWTEYW.QATWIP....EWEFVNT.PPLVKL.....WY..
```

```
PFV-P14350                                TTT                TTT
                                  590       600       610       620
PFV-P14350      ...............SPVKHPSQYEGVFYTDGSAIKSPDPTKSNNAGMGIV
SFV-Mac-P23074  ...............AKTKHPSEFAMVFYTDGSAIKHPDINKSHSAGMGIA
MoMLV-P03355    NCLDILAEAHGTRPDLTDQPLPDADHTWYTDGSSLLQEGQRKAGA......
XMRV-A1Z651     DCLEILAETHGTRPDLTDQPIPDADYTWYTDGSSFLQEGQRRAGA......
HIV-P03366      ...............QLEKEPIVGAETFYVDGAANRETKLGKAGYV.....
```

```
PFV-P14350
                     630       640       650       660       670
PFV-P14350      HATYKPEYQVLNQWSIPLGNHTAQMAEIAAVEFACKKALKIPGPVLVITDS
SFV-Mac-P23074  QVQFQPEYKVIHQWSIPLGDHTAQLAEIAAVEFACKKALKVSGPVLIVTDS
MoMLV-P03355    ..AVTTETEVIWAKALPAGTSA.QRAELIALTQALKM..AEGKKLNVYTDS
XMRV-A1Z651     ..AVTTETEVIWARALPAGTSA.QRAELIALTQALKM..AEGKKLNVYTDS
HIV-P03366      .......TNKGRQKVVPLTNTTNQKTELQAIYLALQD...SGLEVNIVTDS
```

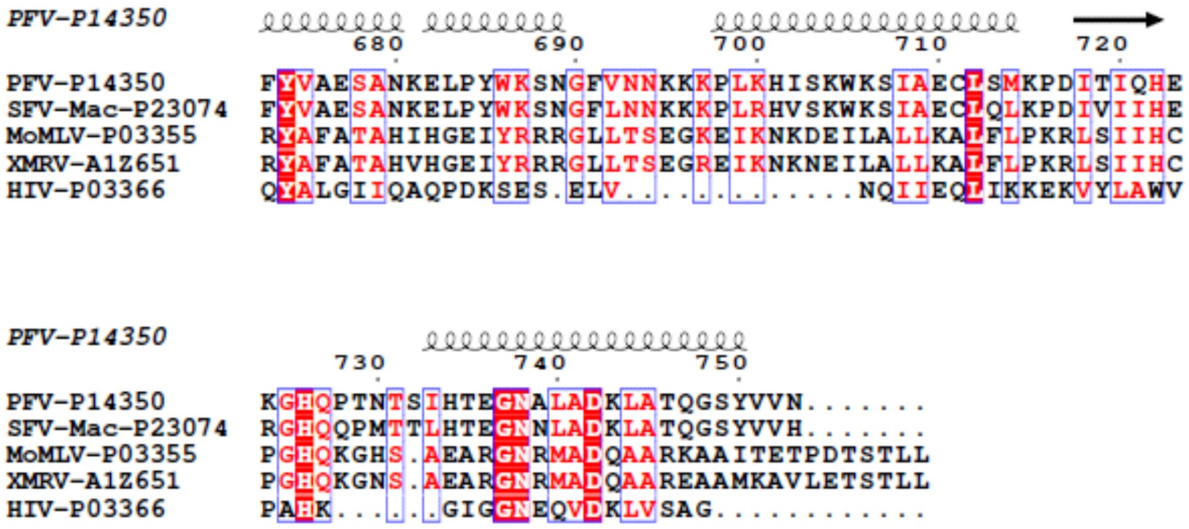

Figure S2. Alignments of selected retroviral PR and RT sequences with PFV PR-RT. Uniprot IDs: PFV- P14350, SFV-P23074, MoMLV- P03355, XMRV- A1Z651, and HIV-P03366. The secondary structures of PFV PR-RT and HIV-1 PR and RT are displayed at the top and bottom of aligned sequences, respectively. The primary sequences were aligned with Clustal Omega and the figure was generated using the program ESPript 3.x. Coiled lines represent α helices, solid arrows represent β strands, and Ts represent β turns.

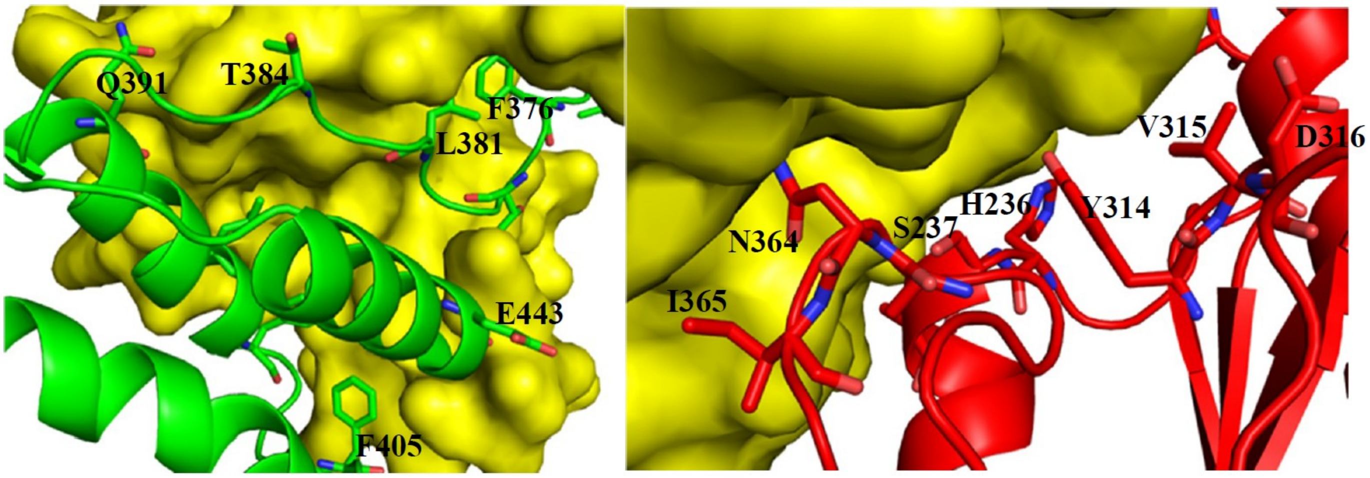

Figure S3. Left panel: interactions at the connection (surface rendered)/thumb (ribbon) interface, Right panel: interactions at the connection (surface rendered)/palm (ribbon) interface.

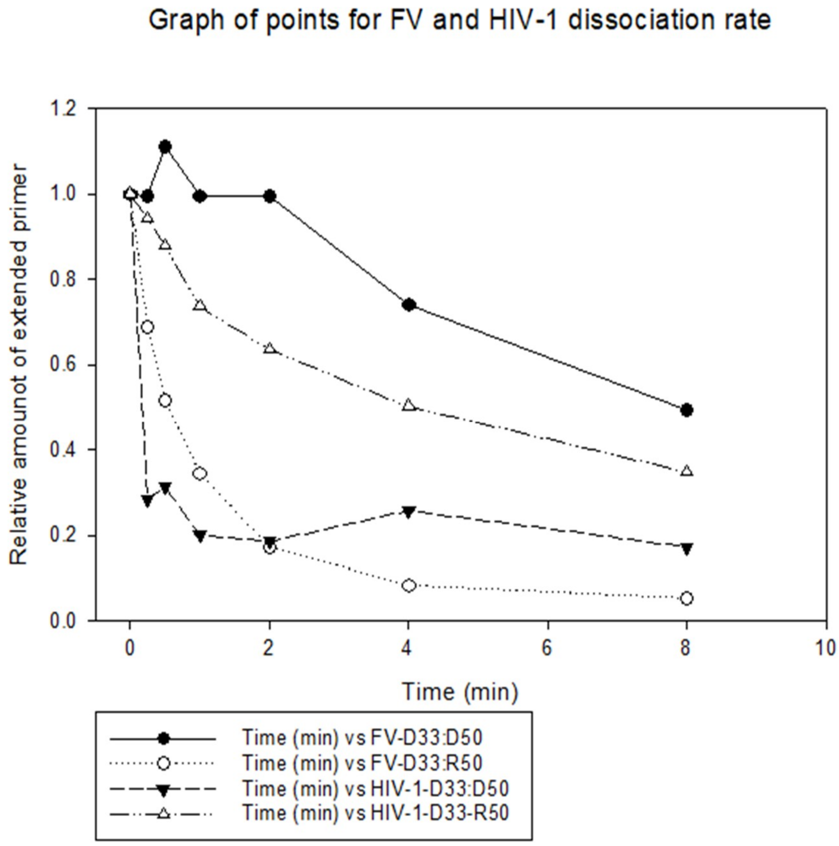

Figure S4. Dissociation rate of PFV PR-RT and HIV-1 RT from an RNA/DNA hybrid (D33-R50) and dsDNA (D33:D50). An off-rate experiment in which HIV RT or WT PFV RT was bound to a 33 nt P-32 5′ end labeled DNA primer (5′-TCCCCGGGTACCGAGCTCGAATTCGCCCTATAG-3′) bound to a 50 nt DNA or RNA template (same sequence: 5′DNA or RNA 5′-TTGTAATACGACTCACTATAGGGCGAATTCGAGCTCGGTACCCGGGGATC-3′) is shown. The bound RT was allowed to dissociate in the presence of a trap to prevent rebinding. Conditions used were 50 mM Tris-HCl, pH 8, 80 mM KCl, 1 mM DTT, 0.1 mM EDTA and 0.1 mg/ml BSA. The "relative amount of extended primer" on the Y-axis represents RT enzyme bound to the hybrid while time is plotted on the X-axis. For detailed methods see: Bohlayer and DeStefano, 2006 (PMID: 16768458).

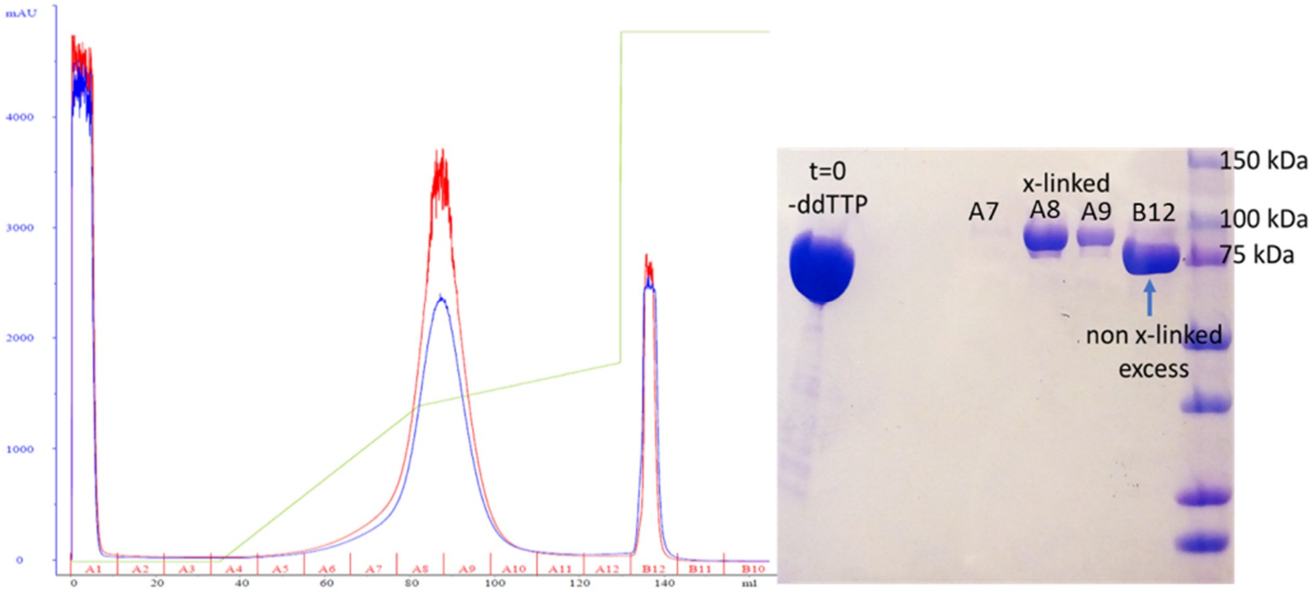

Figure S5. Heparin chromatography trace of PR-RT/dsDNA cross-linked reaction complex with its accompanying SDS-PAGE gel. Elution of the protein was done using a salt gradient.

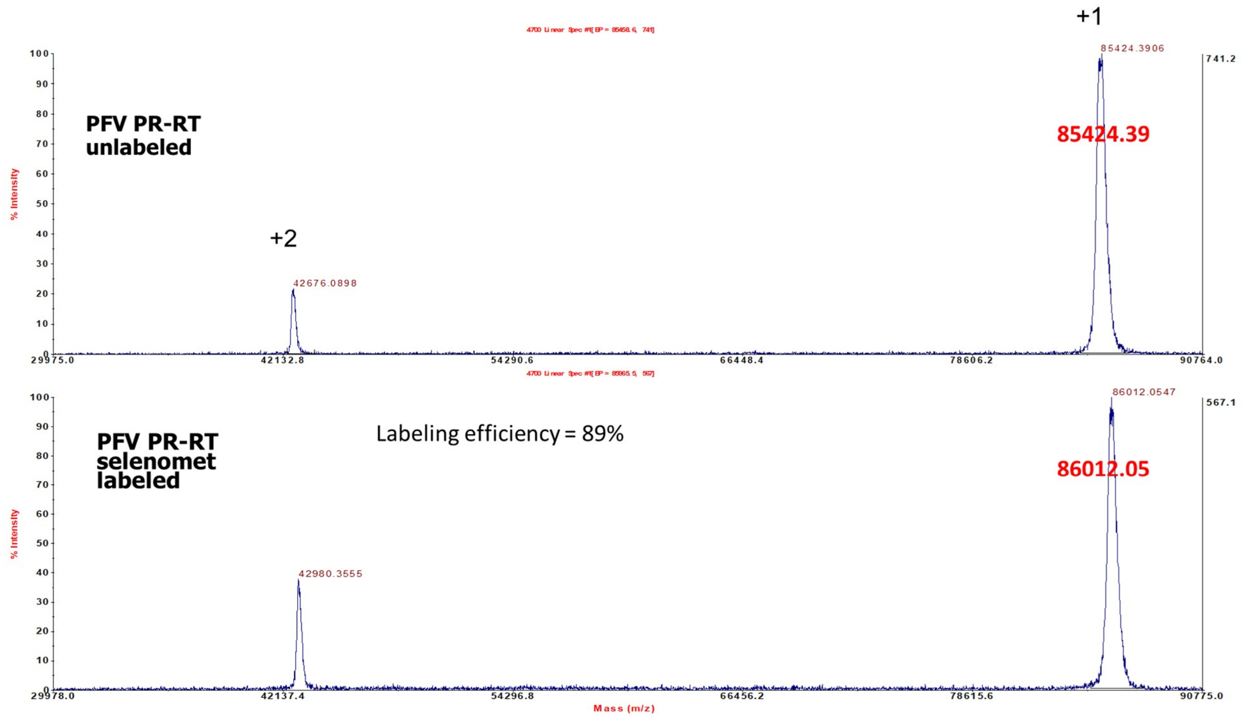

Figure S6. MALDI-TOF mass spectrum of unlabeled and SeMet-labeled PFV PR-RT.

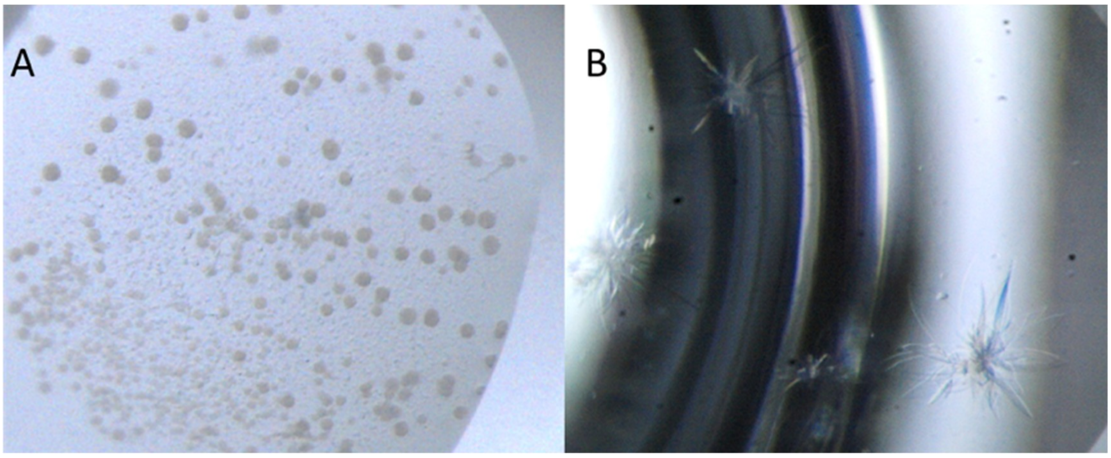

Figure S7. (A) Initial crystallization hit in Natrix HT Screen condition E8 (0.05 M potassium chloride, 0.05 M sodium cacodylate trihydrate pH 6.0, 10% w/v polyethylene glycol 8,000, 0.0005 M spermine, 0.0005 M L-argininamide dihydrochloride). (B) Optimization of initial screening Natrix HT condition E8 using 50 mM EDTA as additive.

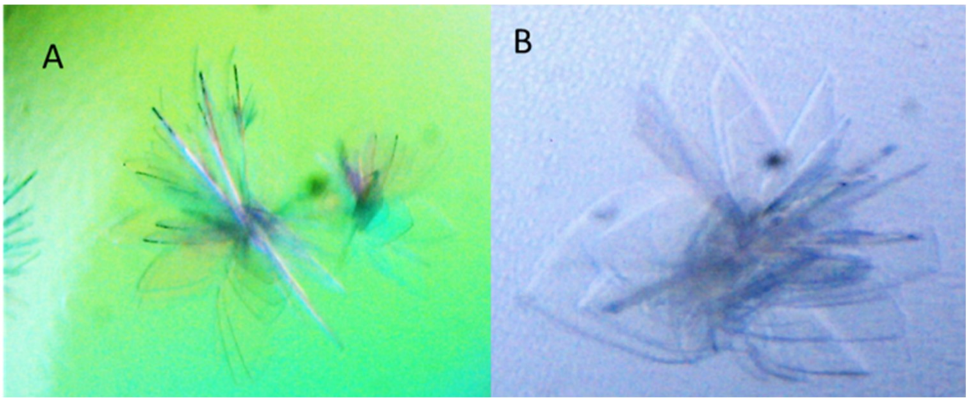

Figure S8. (A and B) Optimized crystals of PFV PR-RT used for structure determination. Optimized crystallization condition: 50 mM KCl, 50 mM sodium cacodylate trihydrate pH 6.0, 12% PEG 8000, 1.0 mM spermine, 1.0 mM L-argininamide, 200 mM glycyglycine or glycylglycylglycine, and 50 mM EDTA (or 10 mM $MgCl_2$, 10 mM $MnCl_2$, or 100 mM $CaCl_2$).

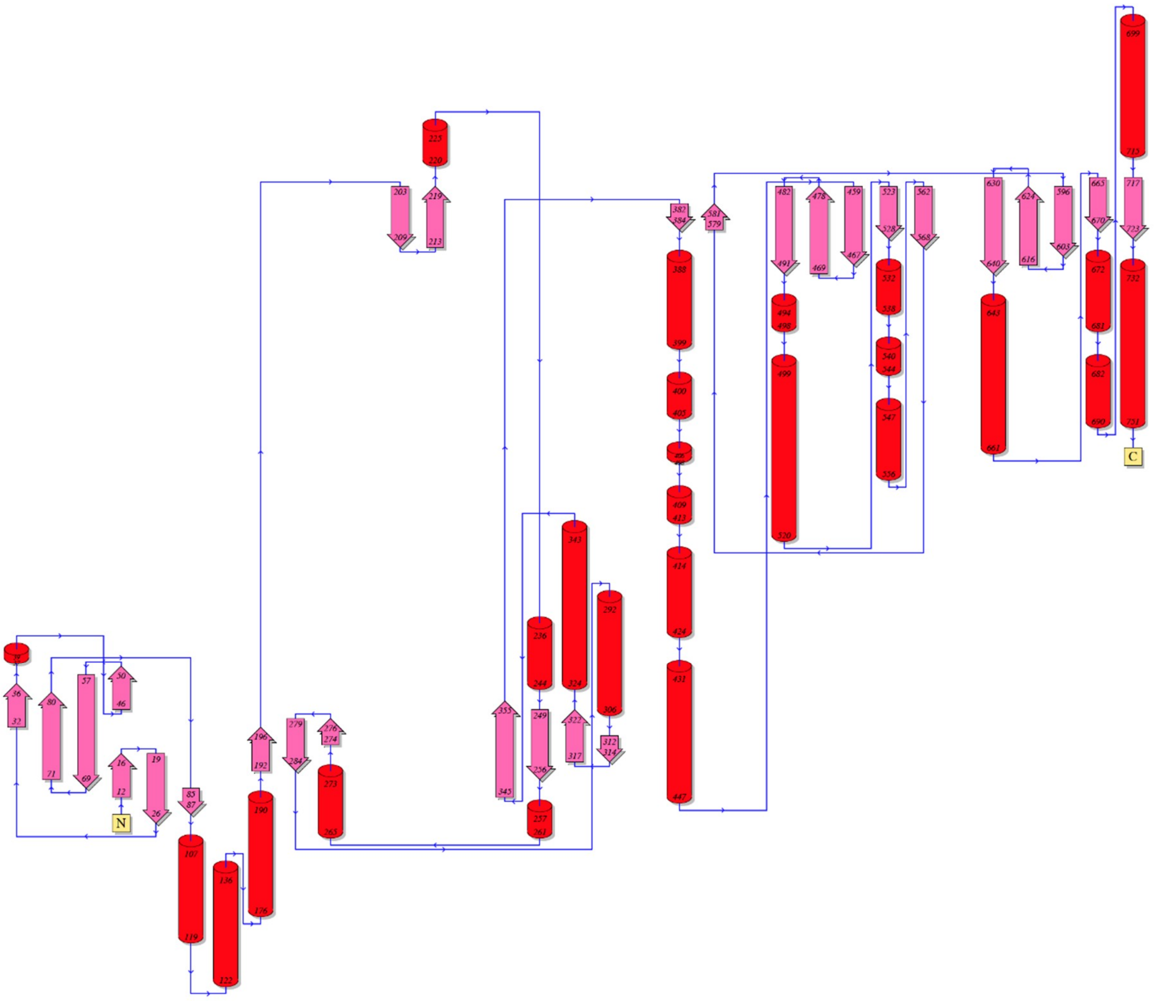

Figure S9. Topological map of the secondary structural elements of the PFV PR-RT.
